# In Silico Analysis of Bioactive Peptides in Invasive Sea Grass *Halophila stipulacea*

**DOI:** 10.3390/cells8060557

**Published:** 2019-06-07

**Authors:** Cagin Kandemir-Cavas, Horacio Pérez-Sanchez, Nazli Mert-Ozupek, Levent Cavas

**Affiliations:** 1 Department of Computer Science, Faculty of Science, Dokuz Eylül University, İzmir 35390, Turkey; cagin.kandemir@deu.edu.tr; 2 Bioinformatics and High Performance Computing Research Group (BIO-HPC), Computer Engineering Department, Universidad Católica de Murcia (UCAM), 30107 Murcia, Spain; 3 Institute of Oncology, Dokuz Eylül University, İzmir 35320, Turkey; nazlimert@hotmail.com.tr; 4 Department of Chemistry, Faculty of Science, Dokuz Eylül University, İzmir 35390, Turkey

**Keywords:** bioactive peptides, *Halophila stipulacea*, in silico analysis, DPP-IV, angiotensin converting enzyme inhibitors

## Abstract

*Halophila stipulacea* is a well-known invasive marine sea grass in the Mediterranean Sea. Having been introduced into the Mediterranean Sea via the Suez Channel, it is considered a Lessepsian migrant. Although, unlike other invasive marine seaweeds, it has not demonstrated serious negative impacts on indigenous species, it does have remarkable invasive properties. The present in-silico study reveals the biotechnological features of *H. stipulacea* by showing bioactive peptides from its rubisc/o protein. These are features such as antioxidant and hypolipideamic activities, dipeptidyl peptidase-IV and angiotensin converting enzyme inhibitions. The reported data open up new applications for such bioactive peptides in the field of pharmacy, medicine and also the food industry.

## 1. Introduction

Invasive species are becoming an important problem in the Mediterranean Sea where many vectors exist, including inflowing water from the Suez Channel and ships’ ballast waters. One of the best-known invasive sea grasses is *Halophila stipulacea* (Forsskål) Ascherson [1], which was first reported in the Mediterranean Sea by Friscth [2]. This sea grass is abundant in the eastern Mediterranean Basin and very common along the Turkish coastline [3]. No validated eradication method for this invasive species is described in the scientific literature. A few reports mention the negative impacts of *H.stipulacea* on indigenous species [4], although Willette and Ambrose [5] mention that little is known of *H. stipulacea*’s effects in its recently discovered Caribbean locations, while Van Tussenbroek et al. [6] report that *H.stipulacea* may be harmful for native species. Although most efforts so far have been devoted to finding alternative ways of evaluating non-indigenous species [7,8], invasive species secrete interesting secondary metabolites that can be exploited economically under the title of *blue biotechnology*. Indeed, recent years have seen growing interest in blue biotechnology-based products [9]. For example, bioactive peptides are one of the candidate targets that can be isolated from invasive species. However, to the best of our knowledge, the bioactive peptides from invasive *H. stipulacea* have not been assessed, and the aim of the present contribution is to fill in this gap in the literature.

Bioactive peptides (hereafter BPs) comprise 3–20 free amino acid food protein fragments [10] composed of covalently bonded (amide/peptide bonds) amino acids [11]. According to the BP database called BIOPEP [12], there are more than 3500 different BPs. The sources of natural BPs can be land-, marine- or food-derived, and include seaweeds [13,14] tropical amphibians [15], cyanobacterium [16], fermented soybean meal [17], sea cucumber [18], cereal crops [19] and milk [20]. BPs are of great importance because of their positive impact on human health. Daliry et al. [21] classified food-derived BPs as anti-cancer, antidiabetic, antihypertensive, antimicrobial, cholesterol-lowering peptides, and multifunctional peptides [21]. Since they help prevent the oxidation and microbial degradation of foods, BPs could be defined as a “new generation” of bioactive regulators [11]. Although BPs are generally coded in the parent protein structure, some BPs are found free in natural sources [11]. Due to their biological and pharmaceutical properties, the production of BPs is of great importance, whether it be by enzymatic hydrolysis [22], chemical synthesis [23] or microbial fermentation [24]. Other production processes include separation and purification techniques such as gel filtration, ultrafiltration [25,26], reverse phase ultra-flow liquid chromatography (RP-UFLC) [25], and reverse phase high performance liquid chromatography (RP-HPLC) and characterization methods such as ultra-performance liquid chromatography tandem mass spectrometry (UPLC-MS/MS) [27]. Since these production, isolation, purification and characterization protocols are time- and solvent-consuming, bioinformatics tools are increasingly used [13,16]. The role of database-aided bioinformatics tools is to quantitatively predict the structure–activity relationship. Many tools have been developed such as BIOPEP [12], Antimicrobial Peptide Database (APD) [28] and PepBank [29]. Ribulose-1,5-bisphosphate carboxylase/oxygenase (RubisCO, E.C. 4.1.1.39) is an important photosynthetic enzyme and the most abundant protein in the world [14,30]. RubisCO is a bifunctional multimeric enzyme and it plays a role in photorespiration and carbon fixation in the Calvin cycle [19,30]. Thirty to fifty percent of RubisCO is soluble and contains eight large (56 kDa) and eight small (14 kDa) subunits [31]. The small subunits of RubisCO contain high amounts of cationic and hydrophobic amino acids [31], while a bioactive dipeptide (Phe-Cys), which suppresses oxidative stress, has been obtained from the large subunit of RubisCO by in-silico thermolysin hydrolysis [32]. Some RubisCO derived peptides have revealed opioid activity, and some are G-protein coupled receptor ligands which constitute the most important class of drug targets [30]. Although there have been attempts at the chemical analysis of *H. stipulacea* [33], its detailed chemical composition is still not known in full.

This contribution presents an alternative and sustainable method for evaluating the invasive sea grass *H. stipulacea* by using in silico analysis of BPs in the large chain of RubisCO. Bioactive peptides are of great importance for the preparation of functional foods because of their excellent health-related effects. Many bioactivities, including antioxidant, antihypertensive and enzyme inhibitory properties have been associated with bioactive peptides from RubisCO of plants [13,19].

## 2. Materials and Methods

### 2.1. Sequence of H. stipulacea Rubisc/o

Ribulose bisphosphate carboxylase large chain of *H. stipulacea* (H6TQS9) was retrieved from the UniProtKB/Swiss-Prot database at the ExPASy Bioinformatics Resource Portal [34]. According to the portal, the sequence provided is a fragment and consists of 200 amino acids.

### 2.2. In silico BIOPEP Parameters

All in-silico calculations were performed using the codes implemented in the BIOPEP webserver [12]. One of the main theoretical parameters (*A*) is defined as the frequency of occurrence of bioactive fragments in the protein chain *A* [12,35,36], which can be calculated by using Equation (1):

(1) A=a/N

where *a* is the number of fragments with given activity in a protein sequence and *N* is the number of amino acid residues in the protein chain [12,35,36].

The frequency with which fragments with given activity were released by enzymes (*A_E_*) and the relative frequency of release of fragments with given activity by enzymes (*W*) were calculated based on Equations (2) and (3), respectively.

(2) $$A_{E}=d/N$$

where *d* is the number of fragments with given activity in the protein sequence that could be released by enzymes, and *N* is the number of amino acid residues in the protein chain. The relative frequency of release of fragments with given activity by selected enzymes (*W*) is given by:

(3) $$W=A_{E}/A$$

The values of *A_E_* and *A* are defined according to Equations (1) and (2), respectively.

Potential biological activity of protein (B) [µM^−1^]:

(4) $$B=\frac{\sum_{i=1}^{k}\frac{a_{i}}{EC_{50i}}}{N}$$

In Equation (4), *a_i_* is the number of repetitions of the *i*-th bioactive fragment in protein sequence, *EC*_50*i*_ is the concentration of the *i*-th bioactive peptide corresponding to its half-maximal activity [µM], *k* is the number of different fragments with given activity and *N* is the number of amino acid residues [35].

The theoretical degree of hydrolysis (*DH_t_*) was also calculated using the following Equation (5):

(5) $$DH_{t}=\frac{d}{D}\times 100\%$$

In Equation (5), *d* is number of hydrolyzed peptide bonds and *D* is total number of peptide bonds in a protein chain.

The relative activity of fragments with given activity released by selected enzymes (*V*) is:

(6) $$V=\frac{B_{E}}{B}$$

In Equation (6), *B_E_* is the activity of fragments potentially released by proteolytic enzyme (enzymes) and *B* is the potential biological activity of the protein.

The amino acid composition of protein was determined based on protein sequences, using the ProtParam program [37] available at [38].

## 3. Results 

*H. stipulacea* large chain RubisCO was retrieved from expasy.org. After in silico proteolytic fragmentation of the RubisCO by BIOPEP tools, bioactive peptides were obtained. The raw data can be found in Appendix A (Table A1, Table A2, Table A3, Table A4, Table A5, Table A6, Table A7, Table A8, Table A9, Table A10, Table A11, Table A12, Table A13, Table A14, Table A15, Table A16, Table A17, Table A18, Table A19, Table A20, Table A21, Table A22, Table A23, Table A24, Table A25, Table A26, Table A27, Table A28, Table A29, Table A30, Table A31, Table A32, Table A33, Table A34, Table A35, Table A36, Table A37, Table A38, Table A39, Table A40, Table A41, Table A42, Table A43, Table A44, Table A45, Table A46, Table A47, Table A48, Table A49, Table A50, Table A51, Table A52, Table A53, Table A54, Table A55 and Table A56). BIOPEP parameters (*A*, *A_E_*, *W*, *BH_t_* and *V*) were extracted from the raw data and the results are presented in the Table 1, Table 2, Table 3, Table 4, Table 5, Table 6, Table 7, Table 8, Table 9 and Table 10.

The angiotensin-converting enzyme (ACE) inhibitor properties of bioactive peptides from RubisCO of *H. stipulacea* were given in Table 1. The values were not calculated for prolyl endopeptidase, clostripain, thrombin, glutamyl endopeptidase II, Xaa-dipeptidase, chymosin, ginger protease (zingipain). From this table, maximum and minimum *A* values were found to be 0.5833 and 0.5808, respectively. The highest values were observed in V-protease, endopeptidase, V-8 protease (Glutamyl endopeptidase), and the minimum values were found in trypsin, plasmin and oligopeptidase B. The maximum *A_E_* value was found to be 0.0874 (calpain 2) and the minimum *A_E_* values were found to be 0.0097 (plasmin, oligopeptidase B, tripsin). The maximum and minimum relative frequency of release of fragments (W) were found if RubisCO is cleaved by calpain 2 (0.1500) and V-protease, glycyl endopeptidase, V8-protease (glutamyl endopeptidase) (0.0084).

The highest *B* value was found to be 0.0055 (subtilisin) and the lowest *B* values were found to be 0 for trypsin, pepsin, plasmin, pancreatic elastase II, oligopeptidase B, glycyl endopeptidase, oligopeptidase F and V-8 protease (Glutamyl endopeptidase). The maximum and minimum *V* values were found to be 0.2057 and 0.0002, respectively. The maximum value was observed in subtilisin and the minimum value was found in glycyl endopeptidase. ACE is known as dipeptidyl carboxypeptidase and one of its major roles is controlling blood pressure [39]. In the literature, Agirbasli and Cavas [13] evaluated the frequency of occurrence (A) values of ACE inhibitor peptides in *Caulerpa* RubisCO and found that *C. racemosa* var. *lamourouxii*, *C. taxifolia* and *C. racemosa f. occidentalis* had the highest *A* values (0.4330, 0.4330 and 0.3993, respectively) and *C. racemosa* var. *turbinata* exhibited the lowest (0.3822). Another study revealed that *C. microphysa* had potential ACE inhibitory activity as a result of pepsin cleavage [40]. However, the number of bioactive peptides in the BIOPEP database has increased, and so the frequency values might have been altered.

The antioxidative properties of BPs from RubisCO of *H. stipulacea* are listed in Table 2. Results reveal that maximum and minimum *A* values were 0.07282 and 0.07280, respectively. The minimum *A* value was obtained when proteinase K was used as protease. Minimum *A* values were found in calpain 2 and proteinase P1 (lactocepin). The maximum and minimum *A_E_* values were obtained as 0.0243 (proteinase K) and 0.0049 (chmytripsin, cathepsin, chymase, papain, ficin, leukocyte elastase, metridin, bromelain and pepsin), respectively. Proteinase K had the maximum *W* value (0.3338) and chymase, papain, ficin, leukocyte elastase, metridin, bromelain and pepsin had the lowest *W* values (0.0673). When thrombin, endopeptidase II, Xaa-Pro dipeptidase, chymosin and ginger protease (zingipain) were used for cleaving, no antioxidative fragment from RubisCO of *H. stipulacea* was found. The inhibition of lipid peroxidation, scavenging of radicals and metal chelation are among the antioxidative properties of BPs [41]. In the literature, the antioxidative activity of BPs have been evaluated in-silico. According to a recent study, the highest *A* value for the antioxidative properties of RubisCO was found in *Caulerpa taxifolia* (0.0785) samples and *C. cylindracea* (0.0759) species [13]. Also, Udenigwe et al. [19,30] found the maximum and minimum *A* value of antioxidative properties of RubisCO of cereal crops to be 0.0568 and 0.0464, respectively [19].

The inhibition effects of bioactive peptides from RubisCO of *H. stipulacea* on dipeptidyl peptidase IV (DPP-IV) (E.C. 3.4.14.5) are given in Table 3. According to the results, prolyl oligopeptidase, V-protease, clostripain and glycyl endopeptidase have the maximum (0.6533) and cathepsin, chymase and metridin have the minimum (0.6497) *A* value. The maximum *A_E_* value was 0.1214 when calpain 2 was used as a protease in DPP-IV inhibitor activity. The minimum *A_E_* value (0.0049) was found in prolyl oligopeptidase, V-protease, clostripain and glycyl endopeptidase. Calpain 2 had the maximum (0.1866) and prolyl oligopeptidase, V-protease and clostripain had the minimum (0.0075) *W* value. We found very low *B* values for all the enzymes studied. The highest *V* value was found in pepsin (pH > 2) (0.4569), while tripsin, prolyl oligopeptidase, V8-protease, plasmin, clostripain, oligopeptidase B, glycyl endopeptidase and proteinase P1 had the lowest *V* value (0.0000). DPP-IV is crucial in glucose metabolism and it degrades the incretins [42]. Thus, DPP-IV inhibitors play a major role in type-2 diabetes mellitus in which insulin secretion and blood glucose level stability are of great importance. [42]. Agirbasli and Cavas found the *A* value of DPP-IV to be between 0.0550 and 0.0714 in all of *Caulerpa* species [13]. They also mentioned that caulerpenyne is found in *Caulerpa* species and its alpha-amylase inhibition activity may play an important role in reducing starch degradation. In another in silico study carried out by Udenigwe et al. [19,30], rice and oat showed the highest *A* value (0.0758).

Table 4 shows the ubiquitin-mediated proteolysis (UbMP) activating properties of bioactive peptides from RubisCO of *H. stipulacea*. The *A* value was 0.0146 in pancreatic elastase, leukocyte elastase, proteinase P1 (lactocepin) and pepsin (pH > 2). The maximum *A_E_* value was 0.0097 (pancreatic elestase) and the minimum *A_E_* values were 0.0049 for leukocyte elastase and proteinase P1. Also, when thrombin, endopeptidase II and Xaa-Pro dipeptidase, chymosin and ginger protease were used, a ubiquitin-mediated proteolysis fragment from *H. stipulacea* was not found. Pancreatic elastase and pepsin have the highest *W* value (0.6644) and leukocyte elastase and proteinase P1 have the lowest *W* value (0.3356). No *B* or *W* value was found for UbMP properties of bioactive peptides from RubisCO of *H. stipulacea*. UbMP is crucial for brain development [43] and its absence causes neurodegenerative diseases such as Parkinson’s and Alzheimer’s [44]. In the literature, Minkiewicz et al. [36] carried out an in-silico evaluation of bovine meat proteins and found that the highest *A* value of activating UbMP was 0.028 in tropomyosin α-1 chain [36].

Results for bioactive regulating fragments from *H. stipulacea* by proteases are shown in Table 5. The results reveal that the only *A* value (0.0146) was found in chymotrypsin, ficin, calpain 2 and pepsin. The maximum *A_E_* value (0.0049) was obtained in chymotrypsin, ficin, calpain 2 and pepsin. Chymotrypsin, ficin, calpain 2 and pepsin had the same *W* values (0.3356). *B* and *V* values were not found for the bioactive regulating activity of *H. stipulacea* by proteases.

Table 6 shows the antithrombotic activity of BPs from *H. stipulacea*. According to the results, maximum *A*, *A_E_* and *W* values (0.0097, 0.0049 and 0.5052, respectively) were obtained for chymotrypsin, calpain 2 and pepsin. The *B* and *V* values of the antithrombotic properties of *H. stipulacea* by proteases were not found. Antithrombic activity is essential for the reduction of thrombin. In the study of Agirbasli and Cavas [13], the *A* values of antithrombic activity of BPs from *Caulerpa* genus were found within the range of 0.0010 to 0.0100 [13].

The antiamnestic activity values of the bioactive peptides are given in Table 7. The only *A* value found (0.0097) was found in chymotrypsin, calpain 2 and pepsin. Also, the maximum *A_E_* and *W* values (0.0049 and 0.5052, respectively) were obtained by chymotrypsin, calpain 2 and pepsin. Agirbasli and Cavas [13] found the *A* values of antiamnestic activity of BPs from *Caulerpa* genus to be within the range of 0.0010 to 0.0100, the same as for antihrombic activity, perhaps because they act in the similar pathway way [13].

The results of stimulating fragments of *H. stipulacea* are given in Table 8. The highest *A*, *A_E_* and *W* values (0.0340, 0.0049 and 0.1441) were obtained by papain and pepsin (pH > 2).

Table 9 provides the immunomodulating activity results of BPs from *H. stipulacea*. The results reveal that calpain 2 has the highest *A*, *A_E_* and *W* values (0.0097, 0.0049 and 0.5052, respectively). It is interesting to note *A*, *A_E_*, *W*, *B* or *V* values could not be calculated for other enzymes in this study.

In Table 10, the theoretical degree of hydrolysis (*DH_t_*) are given for the following proteases: Chymotrypsin A, trypsin, pepsin, proteinase K, pancreatic elastase, prolyl oligopeptidase, V8-protease, thermolysin, chymotrypsin C, plasmin, cathepsin, clostripain, chymase, papain, ficin, leukocyte elastase, metridin, thrombin, pancreatic elastase II, bromelain, endopeptidase II, oligopeptidase B, calpain 2, glycyl endopeptidase, oligopeptidase F, proteinase P1 (lactocepin), Xaa-Pro dipeptidase, pepsin (pH > 2), cocolysin, subtilisin, chymosin, ginger protease (zingipain) and V-8 protease (Glutamyl endopeptidase). According to the results, the highest and the lowest *DH_t_* values were found in pepsin pH > 2 (70.7317) and thrombine, endopeptidase II, Xaa-Pro dipeptidase, chymocine, ginger protease (0), respectively.

In-silico analysis is regarded as an important tool by food scientists since in-silico results may reflect in-vitro and in-vivo results [10,45,46,47,48]. Lafarga et al. [48] defined new bioactive peptides that show ACE and DPP IV inhibition. They confirmed their biological activity by synthetic tripeptides. Sayd et al. [49] also used a similar strategy, grouping the bioactive meat proteins into three categories based on their digestion dynamic. In recent years, there has been a growth in meat consumption as a result of an increasing population. This demand may increase the use of growth hormones, which, in some countries, are banned, but in others allowed [50]. Therefore, an alternative protein source to meat would be of great interest. In this respect, marine seaweeds and seagrasses can be exploited on an industrial scale since there is no hormone ingredient.

## 4. Discussion

Blue biotechnology and blue growth have become two of the hottest topics in recent years. The evaluation of invasive species may open up a new door in the search for novel agents such as vaccines, secondary metabolites and medicines. The present paper reveals that *H.stipulacea* contains bioactive peptides. These peptides can be harvested and evaluated in the countries affected. However, any possible industrial collection of *H.stipulacea* would have to be approved by local authorities. Since *H.stipulacea* forms a mixed vegetation with local Mediterranean macrophytes and seaweeds, its uncontrolled collection might damage the local species. In conclusion, invasive species in the Mediterranean Sea contain very important secondary metabolites and bioactive peptides. Instead of applying blunt eradication methods, biotechnological evaluation is needed.

## Figures and Tables

**Table 1:** The values of the parameters describing the predicted efficiency of bioactive Angiotensin Converting Enzyme (ACE) inhibitor fragment release from *Halophila stipulacea-*RubisCO by proteases.

Proteases	*A*	*A_E_*	*W*	*B*	*V*
Chymotrypsin A	0.5822	0.0485	0.0833	0.0017	0.0645
Trypsin	0.5808	0.0097	0.0167	0.0000	0.0004
Pepsin	0.5826	0.0194	0.0333	0.0000	0.0009
Proteinase K	0.5825	0.0777	0.1334	0.0033	0.1240
Pancreatic elestase	0.5827	0.0437	0.0750	0.0007	0.0256
V-protease	0.5833	0.0049	0.0084	0.0000	0.0003
Thermolysin	0.5825	0.0777	0.1334	0.0052	0.1932
Chymotrypsin C	0.5826	0.0631	0.1083	0.0018	0.0671
Plasmin	0.5808	0.0097	0.0167	0.0000	0.0004
Cathepsin G	0.5826	0.0388	0.0666	0.0008	0.0308
Chymase	0.5826	0.0388	0.0666	0.0008	0.0308
Papain	0.5826	0.0631	0.1083	0.0019	0.0718
Ficin	0.5827	0.0680	0.1167	0.0016	0.0592
Leukocyte elastase	0.5822	0.0485	0.0833	0.0025	0.0916
Metridin	0.5826	0.0388	0.0666	0.0008	0.0308
Pancreatic elastase II	0.5826	0.0194	0.0333	0.0000	0.0009
Bromelain	0.5822	0.0340	0.0584	0.0007	0.0276
Oligopeptidase B	0.5808	0.0097	0.0167	0.0000	0.0004
Calpain 2	0.5827	0.0874	0.1500	0.0034	0.1285
Glycyl endopeptidase	0.5833	0.0049	0.0084	0.0000	0.0002
Oligopeptidase F	0.5826	0.0194	0.0333	0.0000	0.0009
Proteinase P1 (lactocepin)	0.5827	0.0243	0.0417	0.0005	0.0198
Pepsin (pH > 2)	0.5825	0.0777	0.1334	0.0022	0.0828
Coccolysin	0.5827	0.0680	0.1167	0.0021	0.0800
Subtilisin	0.5823	0.0534	0.0917	0.0055	0.2057
V-8 protease (Glutamyl endopeptidase, pH:7.8)	0.5833	0.0049	0.0084	0.0000	0.0003

**Table 2:** The values of parameters describing the predicted efficiency of release of the bioactive antioxidative fragment from *Halophila stipulacea*-RubisCO by proteases.

Proteases	*A*	*A_E_*	*W*	*B*	*V*
Chymotrypsin A	0.0728	0.0049	0.0673	0	0
Proteinase K	0.0728	0.0243	0.3338	0	0
Chymotrypsin C	0.0728	0.0097	0.1332	0	0
Cathepsin	0.0728	0.0049	0.0673	0	0
Chymase	0.0728	0.0049	0.0673	0	0
Papain	0.0728	0.0049	0.0673	0	0
Ficin	0.0728	0.0049	0.0673	0	0
Leukocyte elastase	0.0728	0.0049	0.0673	0	0
Metridin	0.0728	0.0049	0.0673	0	0
Bromelain	0.0728	0.0049	0.0673	0	0
Calpain 2	0.0728	0.0097	0.1332	0	0
Proteinase P1 (lactocepin)	0.0728	0.0097	0.1332	0	0
Pepsin (pH > 2)	0.0728	0.0049	0.0673	0	0
Pubtilisin	0.0728	0.0146	0.2005	0	0

**Table 3:** The values of parameters describing the predicted efficiency of release of bioactive dipeptidyl peptidase IV inhibitor fragment from *Halophila stipulacea*-RubisCO by proteases.

Proteases	*A*	*A_E_*	*W*	*B*	*V*
Chymotrypsin A	0.6501	0.0340	0.0523	0.0000	0.0086
Trypsin	0.6518	0.0146	0.0224	0.0000	0.0000
Pepsin	0.6518	0.0146	0.0224	0.0000	0.0086
Proteinase K	0.6507	0.0922	0.1417	0.0000	0.0345
Pancreatic elestase	0.6508	0.0777	0.1194	0.0000	0.0969
Prolyl oligopeptidase	0.6533	0.0049	0.0075	0.0000	0.0000
V-protease	0.6533	0.0049	0.0075	0.0000	0.0000
Thermolysin	0.6504	0.0534	0.0821	0.0000	0.0071
Chymotrypsin C	0.6501	0.0485	0.0746	0.0000	0.0242
Plasmin	0.6518	0.0146	0.0224	0.0000	0.0000
Cathepsin	0.6497	0.0243	0.0374	0.0000	0.0086
Clostripain	0.6533	0.0049	0.0075	0.0000	0.0000
Chymase	0.6497	0.0243	0.0374	0.0000	0.0086
Papain	0.6506	0.0825	0.1268	0.0000	0.1066
Ficin	0.6503	0.0874	0.1344	0.0000	0.0510
Leukocyte elastase	0.6506	0.0728	0.1119	0.0000	0.1056
Metridin	0.6497	0.0243	0.0374	0.0000	0.0086
Pancreatic elastase II	0.6518	0.0146	0.0224	0.0000	0.0086
Bromelain	0.6507	0.0680	0.1045	0.0000	0.1005
Oligopeptidase B	0.6518	0.0146	0.0224	0.0000	0.0000
Calpain 2	0.6506	0.1214	0.1866	0.0000	0.1066
Glycyl endopeptidase	0.6533	0.0049	0.0075	0.0000	0.0000
Oligopeptidase F	0.6518	0.0146	0.0224	0.0000	0.0086
Proteinase P1 (lactocepin)	0.6501	0.0340	0.0523	0.0000	0.0000
Pepsin (pH > 2)	0.6505	0.1165	0.1791	0.0001	0.4569
Cocolysin	0.6503	0.0437	0.0672	0.0000	0.0071
Subtilisin	0.6507	0.0583	0.0896	0.0000	0.0086
V-8 protease (Glutamyl endopeptidase)	0.6510	0.0097	0.0149	0.0000	0.0000

**Table 4:** The values of parameters describing the predicted efficiency of release of bioactive activating ubiquitin-mediated proteolysis fragment from *Halophila stipulacea*-RubisCO by proteases.

Proteases	*A*	*A_E_*	*W*	*B*	*V*
Pancreatic elastase	0.0146	0.0097	0.6644	0	-
Leukocyte elastase	0.0146	0.0049	0.3356	0	-
Proteinase P1 (lactocepin)	0.0146	0.0049	0.3356	0	-
Pepsin (pH > 2)	0.0146	0.0097	0.6644	0	-

**Table 5:** The values of parameters describing the predicted efficiency of the release of bioactive regulating fragment from *Halophila stipulacea*-RubisCO by proteases.

Proteases	*A*	*A_E_*	*W*	*B*	*V*
Chymotrypsin C	0.0146	0.0049	0.3356	0	-
Ficin	0.0146	0.0049	0.3356	0	-
Calpain 2	0.0146	0.0049	0.3356	0	-
Pepsin (pH > 2)	0.0146	0.0049	0.3356	0	-

**Table 6:** The values of parameters describing the predicted efficiency of release of bioactive antithrombotic fragment from *Halophila stipulacea*-RubisCO by proteases.

Proteases	*A*	*A_E_*	*W*	*B*	*V*
Chymotrypsin C	0.0097	0.0049	0.5052	0	-
Calpain 2	0.0097	0.0049	0.5052	0	-
Pepsin (pH > 2)	0.0097	0.0049	0.5052	0	-

**Table 7:** The values of parameters describing the predicted efficiency of release of bioactive antiamnestic fragment from *Halophila stipulacea*-RubisCO by proteases.

Proteases	*A*	*A_E_*	*W*	*B*	*V*
Chymotrypsin C	0.0097	0.0049	0.5052	0	-
Calpain 2	0.0097	0.0049	0.5052	0	-
Pepsin (pH > 2)	0.0097	0.0049	0.5052	0	-

**Table 8:** The values of parameters describing the predicted efficiency of release of bioactive stimulating fragment from *Halophila stipulacea*-RubisCO by proteases.

Proteases	*A*	*A_E_*	*W*	*B*	*V*
Papain	0.0340	0.0049	0.1441	0	-
Pepsin (pH > 2)	0.0340	0.0049	0.1441	0	-

**Table 9:** The values of parameters describing the predicted efficiency of release of bioactive immunomodulating fragment from *Halophila stipulacea*-RubisCO by proteases.

Proteases	*A*	*A_E_*	*W*	*B*	*V*
Calpain 2	0.0097	0.0049	0.5052	0	0

**Table 10:** Theoretical degree of hydrolysis (*DH_t_*) values for the following enzymes.

Proteases	*DH_t_*
Chymotrypsin A	23.4146
Trypsin	10.7317
Pepsin	12.1951
Proteinase K	37.0732
Pancreatic elastase	54.6341
Prolyl oligopeptidase	6.8293
V-protease	7.3171
Thermolysin	36.5854
Chymotrypsin C	35.6098
Plasmin	10.7317
Cathepsin	20.4878
Clostripain	5.3659
Chymase	19.5122
Papain	43.4146
Ficin	44.3902
Leukocyte elastase	38.5366
Metridin	19.5122
Thrombin	0
Pancreatic elastase II	13.1707
Bromelain	54.1463
Endopeptidase II	0
Oligopeptidase B	10.7317
Calpain 2	47.3171
Glycyl endopeptidase	9.7561
Oligopeptidase F	12.1951
Proteinase P1 (lactocepin)	41.4634
Xaa-Pro dipeptidase	0
Pepsin (pH>2)	70.7317
Cocolysin	30.2439
Subtilisin	29.2683
Chymosin	0
Ginger protease (zingipain)	0
V-8 protease (Glutamyl endopeptidase)	11.7073

## Appendix A. (Raw Data)

**Table A1:** Bioactive peptides from RubisCO of *Halophila stipulacea*. In silico enzymatic cleavage was carried out by using chymotripsine.

No.	Peptide ID	Sequence	Location	Name	Function	Activity	Monoisotopic Mass	Chemical Mass
1	3257	RL	(138–139)	beta-lactokinin	Inhibitor of Angiotensin-Converting Enzyme (ACE) (EC 3.4.15.1) (MEROPS ID: XM02-001)	ACE inhibitor	287.1850	287.3480
2	3384	VF	(128–129)	ACE inhibitor	Inhibitor of Angiotensin-Converting Enzyme (ACE) (EC 3.4.15.1) (MEROPS ID: XM02-001)	ACE inhibitor	264.1360	264.3100
3	3546	VAY	(103–105)	ACE inhibitor	Inhibitor of Angiotensin-Converting Enzyme (ACE) (EC 3.4.15.1) (MEROPS ID: XM02-001)	ACE inhibitor	351.1680	351.3820
4	7513	PL	(106–107)	ACE inhibitor from Alaskan pollack skin	Inhibitor of Angiotensin-converting enzyme (EC 3.4.15.1) (MEROPS ID: XM02-001)	ACE inhibitor	228.1360	228.2770
5	7591	GF	(130–131)	ACE inhibitor	Inhibitor of Angiotensin-Converting Enzyme (ACE) (EC 3.4.15.1) (MEROPS ID: XM02-001)	ACE inhibitor	222.0890	222.2290
6	7599	GL	(183–184)	ACE inhibitor	Inhibitor of Angiotensin-converting enzyme (EC 3.4.15.1) (MEROPS ID: XM02-001)	ACE inhibitor	188.1050	188.2120
7	7691	KY	(168–169)	ACE inhibitor from wakame		ACE inhibitor	309.1570	309.3440
8	7693	KL	(21–22)	ACE inhibitor from wakame		ACE inhibitor	259.1780	259.3340
9	8219	TY	(23–24)	antioxidative peptide		antioxidative	282.1100	282.2750
10	8561	GL	(183–184)	dipeptidyl peptidase IV inhibitor (DPP IV inhibitor)	Inhibitor of Dipeptidyl Peptidase IV (EC 3.4.14.5) (MEROPS ID: S09.003)	dipeptidyl peptidase IV inhibitor	188.1050	188.2120
11	8638	PL	(106–107)	dipeptidyl peptidase IV inhibitor (DPP IV inhibitor)	Inhibitor of Dipeptidyl Peptidase IV (EC 3.4.14.5) (MEROPS ID: S09.003)	dipeptidyl peptidase IV inhibitor	228.1360	228.2770
12	8782	GF	(130–131)	dipeptidyl peptidase IV inhibitor (DPP IV inhibitor)	Inhibitor of Dipeptidyl Peptidase IV (EC 3.4.14.5) (MEROPS ID: S09.003)	dipeptidyl peptidase IV inhibitor	222.0890	222.2290
13	8819	KY	(168–169)	dipeptidyl peptidase IV inhibitor (DPP IV inhibitor)	Inhibitor of Dipeptidyl Peptidase IV (EC 3.4.14.5) (MEROPS ID: S09.003)	dipeptidyl peptidase IV inhibitor	309.1570	309.3440
14	8886	RL	(138–139)	dipeptidyl peptidase IV inhibitor (DPP IV inhibitor)	Inhibitor of Dipeptidyl Peptidase IV (EC 3.4.14.5) (MEROPS ID: S09.003)	dipeptidyl peptidase IV inhibitor	287.1850	287.3480
15	8914	TY	(23–24)	dipeptidyl peptidase IV inhibitor (DPP IV inhibitor)	Inhibitor of Dipeptidyl Peptidase IV (EC 3.4.14.5) (MEROPS ID: S09.003)	dipeptidyl peptidase IV inhibitor	282.1100	282.2750
16	8917	VF	(128–129)	dipeptidyl peptidase IV inhibitor (DPP IV inhibitor)	Inhibitor of Dipeptidyl Peptidase IV (EC 3.4.14.5) (MEROPS ID: S09.003)	dipeptidyl peptidase IV inhibitor	264.1360	264.3100
17	9071	IAY	(100–102)	ACE inhibitor	Inhibitor of Angiotensin-Converting Enzyme (ACE) (EC 3.4.15.1) (MEROPS ID: M02-001)	ACE inhibitor	365.1840	365.4090
18	9074	DF	(204–205)	ACE inhibitor	Inhibitor of Angiotensin-Converting Enzyme (ACE) (EC 3.4.15.1) (MEROPS ID: XM02-001)	ACE inhibitor	280.0949	280.2660

**Table A2:** *A_E_*, *DH_t_*, *W*, *B_E_* and *V* values from RubisCO of *Halophila stipulacea*. In silico enzymatic cleavage carried out by using chymotripsin.

*DH_t_* (%) 23.4146					
No.	Activity	*A_E_*	*W*	*B_E_*	*V*
1	ACE inhibitor	0.0485	0.0833	0.0017261314434853	0.06450702174869
2	antioxidative	0.0049	0.0673	0	0
3	dipeptidyl peptidase IV inhibitor	0.0340	0.0523	1.8563339357632E-6	0.0086054864513139

**Table A3:** Bioactive peptides from RubisCO of *Halophila stipulacea*. In silico enzymatic cleavage was carried out by using trypsine.

No.	Peptide ID	Sequence	Location	Name	Function	Activity	Monoisotopic Mass	Chemical Mass
1	7603	GR	(84–85)	ACE inhibitor	Inhibitor of Angiotensin-Converting Enzyme (ACE) (EC 3.4.15.1) (MEROPS ID: XM02-001)	ACE inhibitor	231.1220	231.2400
2	7697	YK	(82–83)	ACE inhibitor from wakame		ACE inhibitor	309.1570	309.3440
3	8769	DR	(164–165)	dipeptidyl peptidase IV inhibitor (DPP IV inhibitor)	Inhibitor of Dipeptidyl Peptidase IV (EC 3.4.14.5) (MEROPS ID: S09.003)	dipeptidyl peptidase IV inhibitor	289.1279	289.2770
4	8858	PK	(180–181)	dipeptidyl peptidase IV inhibitor (DPP IV inhibitor)	Inhibitor of Dipeptidyl Peptidase IV (EC 3.4.14.5) (MEROPS ID: S09.003)	dipeptidyl peptidase IV inhibitor	243.1460	243.2910
5	8939	YK	(82–83)	dipeptidyl peptidase IV inhibitor (DPP IV inhibitor)	Inhibitor of Dipeptidyl Peptidase IV (EC 3.4.14.5) (MEROPS ID: S09.003)	dipeptidyl peptidase IV inhibitor	309.1570	309.3440

**Table A4:** *A_E_*, *DH_t_*, *W*, *B_E_* and *V* values from RubisCO of *Halophila stipulacea*. In silico enzymatic cleavage carried out by using trypsine.

*DH_t_* (%) 10.7317					
No.	Activity	*A_E_*	*W*	*B_E_*	*V*
1	ACE inhibitor	0.0097	0.0167	9.4749721470635E-6	0.00035408788633428
2	dipeptidyl peptidase IV inhibitor	0.0146	0.0224	0	0

**Table A5:** Bioactive peptides from RubisCO of *Halophila stipulacea*. In silico enzymatic cleavage was carried out by using pepsin.

No.	Peptide ID	Sequence	Location	Name	Function	Activity	Monoisotopic Mass	Chemical Mass
1	3257	RL	(138–139)	beta-lactokinin	Inhibitor of Angiotensin-Converting Enzyme (ACE) (EC 3.4.15.1) (MEROPS ID: XM02-001)	ACE inhibitor	287.1850	287.3480
2	7591	GF	(130–131)	ACE inhibitor	Inhibitor of Angiotensin-Converting Enzyme (ACE) (EC 3.4.15.1) (MEROPS ID: XM02-001)	ACE inhibitor	222.0890	222.2290
3	7599	GL	(183–184)	ACE inhibitor	Inhibitor of Angiotensin-converting enzyme (EC 3.4.15.1) (MEROPS ID: XM02-001)	ACE inhibitor	188.1050	188.2120
4	8561	GL	(183–184)	dipeptidyl peptidase IV inhibitor (DPP IV inhibitor)	Inhibitor of Dipeptidyl Peptidase IV (EC 3.4.14.5) (MEROPS ID: S09.003)	dipeptidyl peptidase IV inhibitor	188.1050	188.2120
5	8782	GF	(130–131)	dipeptidyl peptidase IV inhibitor (DPP IV inhibitor)	Inhibitor of Dipeptidyl Peptidase IV (EC 3.4.14.5) (MEROPS ID: S09.003)	dipeptidyl peptidase IV inhibitor	222.0890	222.2290
6	8886	RL	(138–139)	dipeptidyl peptidase IV inhibitor (DPP IV inhibitor)	Inhibitor of Dipeptidyl Peptidase IV (EC 3.4.14.5) (MEROPS ID: S09.003)	dipeptidyl peptidase IV inhibitor	287.1850	287.3480
7	9074	DF	(204–205)	ACE inhibitor	Inhibitor of Angiotensin-Converting Enzyme (ACE) (EC 3.4.15.1) (MEROPS ID: XM02-001)	ACE inhibitor	280.0949	280.2660

**Table A6:** *A_E_*, *DH_t_*, *W*, *B_E_* and *V* values from RubisCO of *Halophila stipulacea*. In silico enzymatic cleavage carried out by using pepsin.

*DH_t_* (%) 12.1951					
No.	Activity	*A_E_*	*W*	*B_E_*	*V*
1	ACE inhibitor	0.0194	0.0333	2.5006640432763E-5	0.00093451973448837
2	dipeptidyl peptidase IV inhibitor	0.0146	0.0224	1.8563339357632E-6	0.0086054864513139

**Table A7:** Bioactive peptides from RubisCO of *Halophila stipulacea*. In silico enzymatic cleavage was carried out by using proteinase K.

No.	Peptide ID	Sequence	Location	Name	Function	Activity	Monoisotopic Mass	Chemical Mass
1	3257	RL	(138–139)	beta-lactokinin	Inhibitor of Angiotensin-Converting Enzyme (ACE) (EC 3.4.15.1) (MEROPS ID: XM02-001)	ACE inhibitor	287.1850	287.3480
2	3378	GRP	(170–172)	ACE inhibitor	Inhibitor of Angiotensin-Converting Enzyme (ACE) (EC 3.4.15.1) (MEROPS ID: XM02-001)	ACE inhibitor	328.1740	328.3570
3	3563	AY	(101–102)	ACE inhibitor	Inhibitor of Angiotensin-Converting Enzyme (ACE) (EC 3.4.15.1) (MEROPS ID: XM02-001)	ACE inhibitor	252.1000	252.2490
4	3563	AY	(104–105)	ACE inhibitor	Inhibitor of Angiotensin-Converting Enzyme (ACE) (EC 3.4.15.1) (MEROPS ID: XM02-001)	ACE inhibitor	252.1000	252.2490
5	3563	AY	(147–148)	ACE inhibitor	Inhibitor of Angiotensin-Converting Enzyme (ACE) (EC 3.4.15.1) (MEROPS ID: XM02-001)	ACE inhibitor	252.1000	252.2490
6	7591	GF	(12–13)	ACE inhibitor	Inhibitor of Angiotensin-Converting Enzyme (ACE) (EC 3.4.15.1) (MEROPS ID: XM02-001)	ACE inhibitor	222.0890	222.2290
7	7591	GF	(130–131)	ACE inhibitor	Inhibitor of Angiotensin-Converting Enzyme (ACE) (EC 3.4.15.1) (MEROPS ID: XM02-001)	ACE inhibitor	222.0890	222.2290
8	7599	GL	(183–184)	ACE inhibitor	Inhibitor of Angiotensin-converting enzyme (EC 3.4.15.1) (MEROPS ID: XM02-001)	ACE inhibitor	188.1050	188.2120
9	7608	GV	(47–48)	ACE inhibitor	Inhibitor of Angiotensin-Converting Enzyme (ACE) (EC 3.4.15.1) (MEROPS ID: XM02-001)	ACE inhibitor	174.0890	174.1850
10	7693	KL	(21–22)	ACE inhibitor from wakame		ACE inhibitor	259.1780	259.3340
11	7693	KL	(181–182)	ACE inhibitor from wakame		ACE inhibitor	259.1780	259.3340
12	7752	EY	(28–29)	ACE inhibitor from shark meat hydrolysate	Inhibitor of Angiotensin-Converting Enzyme (ACE) (EC 3.4.15.1) (MEROPS ID: XM02-001)	ACE inhibitor	310.1050	310.2860
13	7810	KP	(179–180)	ACE inhibitor from anchovy and bonito	Inhibitor of Angiotensin-Converting Enzyme (ACE) (EC 3.4.15.1) (MEROPS ID: XM02-001)	ACE inhibitor	243.1460	243.2910
14	7866	AY	(101–102)	peptide from Okara protein	Peptide obtained by hydrolysis of Okara protein by use of enzymatic preparation Protease N.	antioxidative	252.1000	252.2490
15	7866	AY	(104–105)	peptide from Okara protein	Peptide obtained by hydrolysis of Okara protein by use of enzymatic preparation Protease N.	antioxidative	252.1000	252.2490
16	7866	AY	(147–148)	peptide from Okara protein	Peptide obtained by hydrolysis of Okara protein by use of enzymatic preparation Protease N.	antioxidative	252.1000	252.2490
17	8218	KP	(179–180)	Antioxidative peptide	Free radical scavenger	antioxidative	243.1460	243.2910
18	8219	TY	(23–24)	antioxidative peptide		antioxidative	282.1100	282.2750
19	8503	TP	(26–27)	Dipeptidyl peptidase IV inhibitor (DPP IV inhibitor)	Inhibitor of Dipeptidyl Peptidase IV (EC 3.4.14.5) (MEROPS ID: S09.003)	dipeptidyl peptidase IV inhibitor	216.0990	216.2220
20	8505	SP	(2–3)	Dipeptidyl peptidase IV inhibitor (DPP IV inhibitor)	Inhibitor of Dipeptidyl Peptidase IV (EC 3.4.14.5) (MEROPS ID: S09.003)	dipeptidyl peptidase IV inhibitor	202.0840	202.1970
21	8505	SP	(43–44)	Dipeptidyl peptidase IV inhibitor (DPP IV inhibitor)	Inhibitor of Dipeptidyl Peptidase IV (EC 3.4.14.5) (MEROPS ID: S09.003)	dipeptidyl peptidase IV inhibitor	202.0840	202.1970
22	8519	KP	(179–180)	dipeptidyl peptidase IV inhibitor (DPP IV inhibitor)	Inhibitor of Dipeptidyl Peptidase IV (EC 3.4.14.5) (MEROPS ID: S09.003)	dipeptidyl peptidase IV inhibitor	243.1460	243.2910
23	8529	EP	(90–91)	dipeptidyl peptidase IV inhibitor (DPP IV inhibitor)	Inhibitor of Dipeptidyl Peptidase IV (EC 3.4.14.5) (MEROPS ID: S09.003)	dipeptidyl peptidase IV inhibitor	244.0940	244.2330
24	8532	QP	(45–46)	dipeptidyl peptidase IV inhibitor (DPP IV inhibitor)	Inhibitor of Dipeptidyl Peptidase IV (EC 3.4.14.5) (MEROPS ID: S09.003)	dipeptidyl peptidase IV inhibitor	243.1100	243.2480
25	8561	GL	(183–184)	dipeptidyl peptidase IV inhibitor (DPP IV inhibitor)	Inhibitor of Dipeptidyl Peptidase IV (EC 3.4.14.5) (MEROPS ID: S09.003)	dipeptidyl peptidase IV inhibitor	188.1050	188.2120
26	8765	AY	(101–102)	dipeptidyl peptidase IV inhibitor (DPP IV inhibitor)	Inhibitor of Dipeptidyl Peptidase IV (EC 3.4.14.5) (MEROPS ID: S09.003)	dipeptidyl peptidase IV inhibitor	252.1000	252.2490
27	8765	AY	(104–105)	dipeptidyl peptidase IV inhibitor (DPP IV inhibitor)	Inhibitor of Dipeptidyl Peptidase IV (EC 3.4.14.5) (MEROPS ID: S09.003)	dipeptidyl peptidase IV inhibitor	252.1000	252.2490
28	8765	AY	(147–148)	dipeptidyl peptidase IV inhibitor (DPP IV inhibitor)	Inhibitor of Dipeptidyl Peptidase IV (EC 3.4.14.5) (MEROPS ID: S09.003)	dipeptidyl peptidase IV inhibitor	252.1000	252.2490
29	8777	EY	(28–29)	dipeptidyl peptidase IV inhibitor (DPP IV inhibitor)	Inhibitor of Dipeptidyl Peptidase IV (EC 3.4.14.5) (MEROPS ID: S09.003)	dipeptidyl peptidase IV inhibitor	310.1050	310.2860
30	8782	GF	(12–13)	dipeptidyl peptidase IV inhibitor (DPP IV inhibitor)	Inhibitor of Dipeptidyl Peptidase IV (EC 3.4.14.5) (MEROPS ID: S09.003)	dipeptidyl peptidase IV inhibitor	222.0890	222.2290
31	8782	GF	(130–131)	dipeptidyl peptidase IV inhibitor (DPP IV inhibitor)	Inhibitor of Dipeptidyl Peptidase IV (EC 3.4.14.5) (MEROPS ID: S09.003)	dipeptidyl peptidase IV inhibitor	222.0890	222.2290
32	8786	GV	(47–48)	dipeptidyl peptidase IV inhibitor (DPP IV inhibitor)	Inhibitor of Dipeptidyl Peptidase IV (EC 3.4.14.5) (MEROPS ID: S09.003)	dipeptidyl peptidase IV inhibitor	174.0890	174.1850
33	8793	HI	(88–89)	dipeptidyl peptidase IV inhibitor (DPP IV inhibitor)	Inhibitor of Dipeptidyl Peptidase IV (EC 3.4.14.5) (MEROPS ID: S09.003)	dipeptidyl peptidase IV inhibitor	268.1420	268.3020
34	8879	QV	(160–161)	dipeptidyl peptidase IV inhibitor (DPP IV inhibitor)	Inhibitor of Dipeptidyl Peptidase IV (EC 3.4.14.5) (MEROPS ID: S09.003)	dipeptidyl peptidase IV inhibitor	245.1260	245.2640
35	8884	RI	(143–144)	dipeptidyl peptidase IV inhibitor (DPP IV inhibitor)	Inhibitor of Dipeptidyl Peptidase IV (EC 3.4.14.5) (MEROPS ID: S09.003)	dipeptidyl peptidase IV inhibitor	287.1850	287.3480
36	8886	RL	(138–139)	dipeptidyl peptidase IV inhibitor (DPP IV inhibitor)	Inhibitor of Dipeptidyl Peptidase IV (EC 3.4.14.5) (MEROPS ID: S09.003)	dipeptidyl peptidase IV inhibitor	287.1850	287.3480
37	8914	TY	(23–24)	dipeptidyl peptidase IV inhibitor (DPP IV inhibitor)	Inhibitor of Dipeptidyl Peptidase IV (EC 3.4.14.5) (MEROPS ID: S09.003)	dipeptidyl peptidase IV inhibitor	282.1100	282.2750
38	9073	TP	(26–27)	ACE inhibitor	Inhibitor of Angiotensin-Converting Enzyme (ACE) (EC 3.4.15.1) (MEROPS ID: M02-001)	ACE inhibitor	216.0990	216.2220
39	9074	DF	(204–205)	ACE inhibitor	Inhibitor of Angiotensin-Converting Enzyme (ACE) (EC 3.4.15.1) (MEROPS ID: XM02-001)	ACE inhibitor	280.0949	280.2660
40	9146	QGP	(153–155)	ACE inhibitor	Inhibitor of Angiotensin-Converting Enzyme (ACE) (EC 3.4.15.1) (MEROPS ID: XM02-001)	ACE inhibitor	300.1310	300.3000

**Table A8:** *A_E_*, *DH_t_*, *W*, *B_E_* and *V* values from RubisCO of *Halophila stipulacea*. In silico enzymatic cleavage was carried out by proteinase K.

*DH_t_* (%) 37.0732					
No.	Activity	*A_E_*	*W*	*B_E_*	*V*
1	ACE inhibitor	0.0777	0.1334	0.0033188734698932	0.12402916586194
2	antioxidative	0.0243	0.3338	0	0
3	dipeptidyl peptidase IV inhibitor	0.0922	0.1417	7.4392944998736E-6	0.034486655009987

**Table A9:** Bioactive peptides from RubisCO of *Halophila stipulacea*. In silico enzymatic cleavage was carried out by using pancreatic elastase.

No.	Peptide ID	Sequence	Location	Name	Function	Activity	Monoisotopic Mass	Chemical Mass
1	3174	KA	(8–9)	dipeptidyl peptidase IV inhibitor (DPP IV inhibitor)	Inhibitor of Dipeptidyl Peptidase IV (EC 3.4.14.5) (MEROPS ID: S09.003)	dipeptidyl peptidase IV inhibitor	217.1310	217.2530
2	3257	RL	(138–139)	beta-lactokinin	Inhibitor of Angiotensin-Converting Enzyme (ACE) (EC 3.4.15.1) (MEROPS ID: XM02-001)	ACE inhibitor	287.1850	287.3480
3	4005	RA	(135–136)		Activation of ubiquitin-dependent proteolysis	activating ubiquitin-mediated proteolysis	245.1380	245.2670
4	4005	RA	(193–194)		Activation of ubiquitin-dependent proteolysis	activating ubiquitin-mediated proteolysis	245.1380	245.2670
5	7513	PL	(106–107)	ACE inhibitor from Alaskan pollack skin	Inhibitor of Angiotensin-converting enzyme (EC 3.4.15.1) (MEROPS ID: XM02-001)	ACE inhibitor	228.1360	228.2770
6	7588	RA	(135–136)	ACE inhibitor	Inhibitor of Angiotensin-Converting Enzyme (ACE) (EC 3.4.15.1) (MEROPS ID: XM02-001)	ACE inhibitor	245.1380	245.2670
7	7588	RA	(193–194)	ACE inhibitor	Inhibitor of Angiotensin-Converting Enzyme (ACE) (EC 3.4.15.1) (MEROPS ID: XM02-001)	ACE inhibitor	245.1380	245.2670
8	7604	KG	(83–84)	ACE inhibitor	Inhibitor of Angiotensin-converting enzyme (EC 3.4.15.1; MEROPS ID: XM02-001)	ACE inhibitor	203.1150	203.2260
9	7605	FG	(129–130)	ACE inhibitor	Inhibitor of Angiotensin-Converting Enzyme (ACE) (EC 3.4.15.1) (MEROPS ID: XM02-001)	ACE inhibitor	222.0890	222.2290
10	7681	DG	(74–75)	ACE inhibitor from soy	Inhibitor of Angiotensin-converting enzyme (ACE) (EC 3.4.15.1) (MEROPS ID: XM02-001)	ACE inhibitor	190.0479	190.1410
11	7693	KL	(21–22)	ACE inhibitor from wakame		ACE inhibitor	259.1780	259.3340
12	7743	KA	(8–9)	ACE inhibitor	Inhibitor of angiotensin-converting enzyme (EC 3.4.15.1) (MEROPS ID: XM02-001)	ACE inhibitor	217.1310	217.2530
13	8526	RA	(135–136)	dipeptidyl peptidase IV inhibitor (DPP IV inhibitor)	Inhibitor of Dipeptidyl Peptidase IV (EC 3.4.14.5) (MEROPS ID: S09.003)	dipeptidyl peptidase IV inhibitor	245.1380	245.2670
14	8526	RA	(193–194)	dipeptidyl peptidase IV inhibitor (DPP IV inhibitor)	Inhibitor of Dipeptidyl Peptidase IV (EC 3.4.14.5) (MEROPS ID: S09.003)	dipeptidyl peptidase IV inhibitor	245.1380	245.2670
15	8638	PL	(106–107)	dipeptidyl peptidase IV inhibitor (DPP IV inhibitor)	Inhibitor of Dipeptidyl Peptidase IV (EC 3.4.14.5) (MEROPS ID: S09.003)	dipeptidyl peptidase IV inhibitor	228.1360	228.2770
16	8685	WT	(68–69)	dipeptidyl peptidase IV inhibitor (DPP IV inhibitor)	Inhibitor of Dipeptidyl Peptidase IV (EC 3.4.14.5) (MEROPS ID: S09.003)	dipeptidyl peptidase IV inhibitor	305.1260	305.3180
17	8685	WT	(72–73)	dipeptidyl peptidase IV inhibitor (DPP IV inhibitor)	Inhibitor of Dipeptidyl Peptidase IV (EC 3.4.14.5) (MEROPS ID: S09.003)	dipeptidyl peptidase IV inhibitor	305.1260	305.3180
18	8774	ET	(6–7)	dipeptidyl peptidase IV inhibitor (DPP IV inhibitor)	Inhibitor of Dipeptidyl Peptidase IV (EC 3.4.14.5) (MEROPS ID: S09.003)	dipeptidyl peptidase IV inhibitor	248.0890	248.2210
19	8774	ET	(30–31)	dipeptidyl peptidase IV inhibitor (DPP IV inhibitor)	Inhibitor of Dipeptidyl Peptidase IV (EC 3.4.14.5) (MEROPS ID: S09.003)	dipeptidyl peptidase IV inhibitor	248.0890	248.2210
20	8793	HI	(88–89)	dipeptidyl peptidase IV inhibitor (DPP IV inhibitor)	Inhibitor of Dipeptidyl Peptidase IV (EC 3.4.14.5) (MEROPS ID: S09.003)	dipeptidyl peptidase IV inhibitor	268.1420	268.3020
21	8810	KG	(83–84)	dipeptidyl peptidase IV inhibitor (DPP IV inhibitor)	Inhibitor of Dipeptidyl Peptidase IV (EC 3.4.14.5) (MEROPS ID: S09.003)	dipeptidyl peptidase IV inhibitor	203.1150	203.2260
22	8816	KT	(150–151)	dipeptidyl peptidase IV inhibitor (DPP IV inhibitor)	Inhibitor of Dipeptidyl Peptidase IV (EC 3.4.14.5) (MEROPS ID: S09.003)	dipeptidyl peptidase IV inhibitor	247.1410	247.2790
23	8851	NV	(127–128)	dipeptidyl peptidase IV inhibitor (DPP IV inhibitor)	Inhibitor of Dipeptidyl Peptidase IV (EC 3.4.14.5) (MEROPS ID: S09.003)	dipeptidyl peptidase IV inhibitor	231.1109	231.2370
24	8879	QV	(160–161)	dipeptidyl peptidase IV inhibitor (DPP IV inhibitor)	Inhibitor of Dipeptidyl Peptidase IV (EC 3.4.14.5) (MEROPS ID: S09.003)	dipeptidyl peptidase IV inhibitor	245.1260	245.2640
25	8882	RG	(200–201)	dipeptidyl peptidase IV inhibitor (DPP IV inhibitor)	Inhibitor of Dipeptidyl Peptidase IV (EC 3.4.14.5) (MEROPS ID: S09.003)	dipeptidyl peptidase IV inhibitor	231.1220	231.2400
26	8884	RI	(143–144)	dipeptidyl peptidase IV inhibitor (DPP IV inhibitor)	Inhibitor of Dipeptidyl Peptidase IV (EC 3.4.14.5) (MEROPS ID: S09.003)	dipeptidyl peptidase IV inhibitor	287.1850	287.3480
27	8886	RL	(138–139)	dipeptidyl peptidase IV inhibitor (DPP IV inhibitor)	Inhibitor of Dipeptidyl Peptidase IV (EC 3.4.14.5) (MEROPS ID: S09.003)	dipeptidyl peptidase IV inhibitor	287.1850	287.3480

**Table A10:** *A_E_*, *DH_t_*, *W*, *B_E_* and *V* values from RubisCO of *Halophila stipulacea*. In silico enzymatic cleavage was carried out by pancreatic elastase.

*DH_t_* (%) 54.6341					
No.	Activity	*A_E_*	*W*	*B_E_*	*V*
1	dipeptidyl peptidase IV inhibitorinhibitor	0.0777	0.1194	2.0912653098933E-5	0.096945678488557
2	ACE inhibitor	0.0437	0.0750	0.0006857879028624	0.025628485786464
3	activating ubiquitin-mediated proteolysis	0.0097	0.6644	0	

**Table A11:** Bioactive peptides from RubisCO of *Halophila stipulacea*. In silico enzymatic cleavage was carried out by using prolyl oligopeptidase.

No.	Peptide ID	Sequence	Location	Name	Function	Activity	Monoisotopic Mass	Chemical Mass
1	8532	QP	(45–46)	dipeptidyl peptidase IV inhibitor (DPP IV inhibitor)	Inhibitor of Dipeptidyl Peptidase IV (EC 3.4.14.5) (MEROPS ID: S09.003)	dipeptidyl peptidase IV inhibitor	243.1100	243.2480

**Table A12:** *A_E_*, *DH_t_*, *W*, *B_E_* and *V* values from RubisCO of *Halophila stipulacea*. In silico enzymatic cleavage was carried out by prolyl oligopeptidase.

*DH_t_* (%) 6.8293					
No.	Activity	*A_E_*	*W*	*B_E_*	*V*
1	dipeptidyl peptidase IV inhibitor	0.0049	0.0075	0	0

**Table A13:** Bioactive peptides from RubisCO of *Halophila stipulacea*. In silico enzymatic cleavage was carried out by using V-protease.

No.	Peptide ID	Sequence	Location	Name	Function	Activity	Monoisotopic Mass	Chemical Mass
1	8934	YE	(29–30)	dipeptidyl peptidase IV inhibitor (DPP IV inhibitor)	Inhibitor of Dipeptidyl Peptidase IV (EC 3.4.14.5) (MEROPS ID: S09.003)	dipeptidyl peptidase IV inhibitor	310.1050	310.2860
2	9078	YE	(29–30)	ACE inhibitor	Inhibitor of Angiotensin-Converting Enzyme (ACE) (EC 3.4.15.1) (MEROPS ID: XM02-001)	ACE inhibitor	310.1050	310.2860

**Table A14:** *A_E_*, *DH_t_*, *W*, *B_E_* and *V* values from RubisCO of *Halophila stipulacea*. In silico enzymatic cleavage was carried out by V-protease.

*DH_t_* (%) 7.3171					
No.	Activity	*A_E_*	*W*	*B_E_*	*V*
1	dipeptidyl peptidase IV inhibitor	0.0049	0.0075	0	0
2	ACE inhibitor	0.0049	0.0084	7.6943555746376E-6	0.0002875447082947

**Table A15:** Bioactive peptides from RubisCO of *Halophila stipulacea*. In silico enzymatic cleavage was carried out by using thermolysin.

No.	Peptide ID	Sequence	Location	Name	Function	Activity	Monoisotopic Mass	Chemical Mass
1	3522	IPP	(144–146)	ACE inhibitor (from bovine b-CN)		ACE inhibitor	325.1880	325.3940
2	3666	YP	(105–106)	ACE inhibitor	Inhibitor of Angiotensin-Converting Enzyme (ACE) (EC 3.4.15.1) (MEROPS ID: XM02-001)	ACE inhibitor	278.1150	278.2870
3	7592	FR	(40–41)	ACE inhibitor		ACE inhibitor	321.1690	321.3650
4	7594	VG	(11–12)	ACE inhibitor	Inhibitor of Angiotensin-Converting Enzyme (ACE) (EC 3.4.15.1) (MEROPS ID: XM02-001)	ACE inhibitor	174.0890	174.1850
5	7600	AG	(9–10)	ACE inhibitor	Inhibitor of Angiotensin-Converting Enzyme (ACE) (EC 3.4.15.1) (MEROPS ID: XM02-001)	ACE inhibitor	146.0580	146.1310
6	7600	AG	(15–16)	ACE inhibitor	Inhibitor of Angiotensin-Converting Enzyme (ACE) (EC 3.4.15.1) (MEROPS ID: XM02-001)	ACE inhibitor	146.0580	146.1310
7	7600	AG	(53–54)	ACE inhibitor	Inhibitor of Angiotensin-Converting Enzyme (ACE) (EC 3.4.15.1) (MEROPS ID: XM02-001)	ACE inhibitor	146.0580	146.1310
8	7605	FG	(129–130)	ACE inhibitor	Inhibitor of Angiotensin-Converting Enzyme (ACE) (EC 3.4.15.1) (MEROPS ID: XM02-001)	ACE inhibitor	222.0890	222.2290
9	7619	LG	(182–183)	ACE inhibitor	Inhibitor of Angiotensin-converting enzyme (EC 3.4.15.1) (MEROPS ID: XM02-001)	ACE inhibitor	188.1050	188.2120
10	7697	YK	(20–21)	ACE inhibitor from wakame		ACE inhibitor	309.1570	309.3440
11	7859	IEP	(89–91)	ACE inhibitor	Inhibitor of Angiotensin-Converting Enzyme (ACE) (EC 3.4.15.1) (MEROPS ID: XM02-001)	ACE inhibitor	357.1780	357.3930
12	8521	YP	(105–106)	dipeptidyl peptidase IV inhibitor (DPP IV inhibitor)	Inhibitor of Dipeptidyl Peptidase IV (EC 3.4.14.5) (MEROPS ID: S09.003)	dipeptidyl peptidase IV inhibitor	278.1150	278.2870
13	8760	AG	(9–10)	dipeptidyl peptidase IV inhibitor (DPP IV inhibitor)	Inhibitor of Dipeptidyl Peptidase IV (EC 3.4.14.5) (MEROPS ID: S09.003)	dipeptidyl peptidase IV inhibitor	146.0580	146.1310
14	8760	AG	(15–16)	dipeptidyl peptidase IV inhibitor (DPP IV inhibitor)	Inhibitor of Dipeptidyl Peptidase IV (EC 3.4.14.5) (MEROPS ID: S09.003)	dipeptidyl peptidase IV inhibitor	146.0580	146.1310
15	8760	AG	(53–54)	dipeptidyl peptidase IV inhibitor (DPP IV inhibitor)	Inhibitor of Dipeptidyl Peptidase IV (EC 3.4.14.5) (MEROPS ID: S09.003)	dipeptidyl peptidase IV inhibitor	146.0580	146.1310
16	8779	FQ	(13–14)	dipeptidyl peptidase IV inhibitor (DPP IV inhibitor)	Inhibitor of Dipeptidyl Peptidase IV (EC 3.4.14.5) (MEROPS ID: S09.003)	dipeptidyl peptidase IV inhibitor	293.1260	293.3080
17	8780	FR	(40–41)	dipeptidyl peptidase IV inhibitor (DPP IV inhibitor)	Inhibitor of Dipeptidyl Peptidase IV (EC 3.4.14.5) (MEROPS ID: S09.003)	dipeptidyl peptidase IV inhibitor	321.1690	321.3650
18	8805	IQ	(159–160)	dipeptidyl peptidase IV inhibitor (DPP IV inhibitor)	Inhibitor of Dipeptidyl Peptidase IV (EC 3.4.14.5) (MEROPS ID: S09.003)	dipeptidyl peptidase IV inhibitor	259.1420	259.2910
19	8824	LT	(22–23)	dipeptidyl peptidase IV inhibitor (DPP IV inhibitor)	Inhibitor of Dipeptidyl Peptidase IV (EC 3.4.14.5) (MEROPS ID: S09.003)	dipeptidyl peptidase IV inhibitor	232.1310	232.2650
20	8918	VG	(11–12)	dipeptidyl peptidase IV inhibitor (DPP IV inhibitor)	Inhibitor of Dipeptidyl Peptidase IV (EC 3.4.14.5) (MEROPS ID: S09.003)	dipeptidyl peptidase IV inhibitor	174.0890	174.1850
21	8937	YH	(87–88)	dipeptidyl peptidase IV inhibitor (DPP IV inhibitor)	Inhibitor of Dipeptidyl Peptidase IV (EC 3.4.14.5) (MEROPS ID: S09.003)	dipeptidyl peptidase IV inhibitor	318.1210	318.3120
22	8939	YK	(20–21)	dipeptidyl peptidase IV inhibitor (DPP IV inhibitor)	Inhibitor of Dipeptidyl Peptidase IV (EC 3.4.14.5) (MEROPS ID: S09.003)	dipeptidyl peptidase IV inhibitor	309.1570	309.3440
23	9076	FQ	(13–14)	ACE inhibitor	Inhibitor of Angiotensin-Converting Enzyme (ACE) (EC 3.4.15.1) (MEROPS ID: XM02-001)	ACE inhibitor	293.1260	293.3080
24	9087	YH	(87–88)	ACE inhibitor	Inhibitor of Angiotensin-Converting Enzyme (ACE) (EC 3.4.15.1) (MEROPS ID: XM02-001)	ACE inhibitor	318.1210	318.3120
25	9213	LR	(134–135)	ACE inhibitor	Inhibitor of Angiotensin-Converting Enzyme (ACE) (EC 3.4.15.1) (MEROPS ID: M02-001)	ACE inhibitor	287.1850	287.3480
26	9213	LR	(137–138)	ACE inhibitor	Inhibitor of Angiotensin-Converting Enzyme (ACE) (EC 3.4.15.1) (MEROPS ID: M02-001)	ACE inhibitor	287.1850	287.3480
27	9213	LR	(142–143)	ACE inhibitor	Inhibitor of Angiotensin-Converting Enzyme (ACE) (EC 3.4.15.1) (MEROPS ID: M02-001)	ACE inhibitor	287.1850	287.3480

**Table A16:** *A_E_*, *DH_t_*, *W*, *B_E_* and *V* values from RubisCO of *Halophila stipulacea*. In silico enzymatic cleavage was carried out by thermolysin.

*DH_t_* (%) 36.5854					
No.	Activity	*A_E_*	*W*	*B_E_*	*V*
1	ACE inhibitor	0.0777	0.1334	0.0051700207807555	0.19320813846713
2	dipeptidyl peptidase IV inhibitor	0.0534	0.0821	1.5313466662583E-6	0.0070989290961449

**Table A17:** Bioactive peptides from RubisCO of *Halophila stipulacea*. In silico enzymatic cleavage was carried out by using Chymotrypsin.

No.	Peptide ID	Sequence	Location	Name	Function	Activity	Monoisotopic Mass	Chemical Mass
1	2753	GP	(154–155)	peptide regulating the stomach mucosal membrane activity	regulating the stomach mucosal membrane activity	regulating	172.0730	172.1690
2	3169	GP	(154–155)	dipeptidyl peptidase IV inhibitor (DPP IV inhibitor)	Inhibitor of Dipeptidyl Peptidase IV (EC 3.4.14.5) (MEROPS ID: S09.003)	dipeptidyl peptidase IV inhibitor	172.0730	172.1690
3	3257	RL	(138–139)	beta-lactokinin	Inhibitor of Angiotensin-Converting Enzyme (ACE) (EC 3.4.15.1) (MEROPS ID: XM02-001)	ACE inhibitor	287.1850	287.3480
4	3283	GP	(154–155)		antithrombotic	antithrombotic	172.0730	172.1690
5	3378	GRP	(170–172)	ACE inhibitor	Inhibitor of Angiotensin-Converting Enzyme (ACE) (EC 3.4.15.1) (MEROPS ID: XM02-001)	ACE inhibitor	328.1740	328.3570
6	3461	GP	(154–155)	Prolyl endopeptidase inhibitor	Inhibitor of Prolyl Endopeptidase (PEP) (EC 3.4.21.26) (MEROPS ID: S09.001)	antiamnestic	172.0730	172.1690
7	3546	VAY	(103–105)	ACE inhibitor	Inhibitor of Angiotensin-Converting Enzyme (ACE) (EC 3.4.15.1) (MEROPS ID: XM02-001)	ACE inhibitor	351.1680	351.3820
8	3563	AY	(147–148)	ACE inhibitor	Inhibitor of Angiotensin-Converting Enzyme (ACE) (EC 3.4.15.1) (MEROPS ID: XM02-001)	ACE inhibitor	252.1000	252.2490
9	7512	GP	(154–155)	ACE inhibitor from Alaskan pollack skin	Inhibitor of Angiotensin-Converting Enzyme (ACE) (EC 3.4.15.1) (MEROPS ID: XM02-001)	ACE inhibitor	172.0730	172.1690
10	7599	GL	(183–184)	ACE inhibitor	Inhibitor of Angiotensin-converting enzyme (EC 3.4.15.1) (MEROPS ID: XM02-001)	ACE inhibitor	188.1050	188.2120
11	7691	KY	(168–169)	ACE inhibitor from wakame		ACE inhibitor	309.1570	309.3440
12	7693	KL	(21–22)	ACE inhibitor from wakame		ACE inhibitor	259.1780	259.3340
13	7693	KL	(181–182)	ACE inhibitor from wakame		ACE inhibitor	259.1780	259.3340
14	7829	VE	(161–162)	ACE inhibitor	Inhibitor of Angiotensin-Converting Enzyme (ACE) (EC 3.4.15.1) (MEROPS ID: XM02-001)	ACE inhibitor	246.1100	246.2490
15	7830	TE	(5–6)	ACE inhibitor	Inhibitor of Angiotensin-Converting Enzyme (EC 3.4.15.1) (MEROPS ID: XM02-001)	ACE inhibitor	248.0890	248.2210
16	7866	AY	(147–148)	peptide from Okara protein	Peptide obtained by hydrolysis of Okara protein by use of enzymatic preparation Protease N.	antioxidative	252.1000	252.2490
17	8219	TY	(23–24)	antioxidative peptide		antioxidative	282.1100	282.2750
18	8503	TP	(26–27)	Dipeptidyl peptidase IV inhibitor (DPP IV inhibitor)	Inhibitor of Dipeptidyl Peptidase IV (EC 3.4.14.5) (MEROPS ID: S09.003)	dipeptidyl peptidase IV inhibitor	216.0990	216.2220
19	8505	SP	(2–3)	Dipeptidyl peptidase IV inhibitor (DPP IV inhibitor)	Inhibitor of Dipeptidyl Peptidase IV (EC 3.4.14.5) (MEROPS ID: S09.003)	dipeptidyl peptidase IV inhibitor	202.0840	202.1970
20	8561	GL	(183–184)	dipeptidyl peptidase IV inhibitor (DPP IV inhibitor)	Inhibitor of Dipeptidyl Peptidase IV (EC 3.4.14.5) (MEROPS ID: S09.003)	dipeptidyl peptidase IV inhibitor	188.1050	188.2120
21	8765	AY	(147–148)	dipeptidyl peptidase IV inhibitor (DPP IV inhibitor)	Inhibitor of Dipeptidyl Peptidase IV (EC 3.4.14.5) (MEROPS ID: S09.003)	dipeptidyl peptidase IV inhibitor	252.1000	252.2490
22	8819	KY	(168–169)	dipeptidyl peptidase IV inhibitor (DPP IV inhibitor)	Inhibitor of Dipeptidyl Peptidase IV (EC 3.4.14.5) (MEROPS ID: S09.003)	dipeptidyl peptidase IV inhibitor	309.1570	309.3440
23	8886	RL	(138–139)	dipeptidyl peptidase IV inhibitor (DPP IV inhibitor)	Inhibitor of Dipeptidyl Peptidase IV (EC 3.4.14.5) (MEROPS ID: S09.003)	dipeptidyl peptidase IV inhibitor	287.1850	287.3480
24	8899	TE	(5–6)	dipeptidyl peptidase IV inhibitor (DPP IV inhibitor)	Inhibitor of Dipeptidyl Peptidase IV (EC 3.4.14.5) (MEROPS ID: S09.003)	dipeptidyl peptidase IV inhibitor	248.0890	248.2210
25	8914	TY	(23–24)	dipeptidyl peptidase IV inhibitor (DPP IV inhibitor)	Inhibitor of Dipeptidyl Peptidase IV (EC 3.4.14.5) (MEROPS ID: S09.003)	dipeptidyl peptidase IV inhibitor	282.1100	282.2750
26	8916	VE	(161–162)	dipeptidyl peptidase IV inhibitor (DPP IV inhibitor)	Inhibitor of Dipeptidyl Peptidase IV (EC 3.4.14.5) (MEROPS ID: S09.003)	dipeptidyl peptidase IV inhibitor	246.1100	246.2490
27	9071	IAY	(100–102)	ACE inhibitor	Inhibitor of Angiotensin-Converting Enzyme (ACE) (EC 3.4.15.1) (MEROPS ID: M02-001)	ACE inhibitor	365.1840	365.4090
28	9073	TP	(26–27)	ACE inhibitor	Inhibitor of Angiotensin-Converting Enzyme (ACE) (EC 3.4.15.1) (MEROPS ID: M02-001)	ACE inhibitor	216.0990	216.2220

**Table A18:** *A_E_*, *DH_t_*, *W*, *B_E_* and *V* values from RubisCO of *Halophila stipulacea*. In silico enzymatic cleavage was carried out by Chymotrypsin.

*DH_t_* (%) 35.6098					
No.	Activity	*A_E_*	*W*	*B_E_*	*V*
1	regulating	0.0049	0.3356	0	
2	dipeptidyl peptidase IV inhibitor	0.0485	0.0746	5.217325115104E-6	0.024186176702991
3	ACE inhibitor	0.0631	0.1083	0.0017951962898486	0.067088034662415
4	antithrombotic	0.0049	0.5052	0	
5	antiamnestic	0.0049	0.5052	0	
6	antioxidative	0.0097	0.1332	0	0

**Table A19:** Bioactive peptides from RubisCO of *Halophila stipulacea*. In silico enzymatic cleavage was carried out by using plasmin.

No.	Peptide ID	Sequence	Location	Name	Function	Activity	Monoisotopic Mass	Chemical Mass
1	7603	GR	(84–85)	ACE inhibitor	Inhibitor of Angiotensin-Converting Enzyme (ACE) (EC 3.4.15.1) (MEROPS ID: XM02-001)	ACE inhibitor	231.1220	231.2400
2	7697	YK	(82–83)	ACE inhibitor from wakame		ACE inhibitor	309.1570	309.3440
3	8769	DR	(164–165)	dipeptidyl peptidase IV inhibitor (DPP IV inhibitor)	Inhibitor of Dipeptidyl Peptidase IV (EC 3.4.14.5) (MEROPS ID: S09.003)	dipeptidyl peptidase IV inhibitor	289.1279	289.2770
4	8858	PK	(180–181)	dipeptidyl peptidase IV inhibitor (DPP IV inhibitor)	Inhibitor of Dipeptidyl Peptidase IV (EC 3.4.14.5) (MEROPS ID: S09.003)	dipeptidyl peptidase IV inhibitor	243.1460	243.2910
5	8939	YK	(82–83)	dipeptidyl peptidase IV inhibitor (DPP IV inhibitor)	Inhibitor of Dipeptidyl Peptidase IV (EC 3.4.14.5) (MEROPS ID: S09.003)	dipeptidyl peptidase IV inhibitor	309.1570	309.3440

**Table A20:** *A_E_*, *DH_t_*, *W*, *B_E_* and *V* values from RubisCO of *Halophila stipulacea*. In silico enzymatic cleavage was carried out by plasmin.

*DH_t_* (%) 10.7317					
No.	Activity	*A_E_*	*W*	*B_E_*	*V*
1	ACE inhibitor	0.0097	0.0167	9.4749721470635E-6	0.00035408788633428
2	dipeptidyl peptidase IV inhibitor	0.0146	0.0224	0	0

**Table A21:** Bioactive peptides from RubisCO of *Halophila stipulacea*. In silico enzymatic cleavage was carried out by using cathepsin.

No.	Peptide ID	Sequence	Location	Name	Function	Activity	Monoisotopic Mass	Chemical Mass
1	3257	RL	(138–139)	beta-lactokinin	Inhibitor of Angiotensin-Converting Enzyme (ACE) (EC 3.4.15.1) (MEROPS ID: XM02-001)	ACE inhibitor	287.1850	287.3480
2	3546	VAY	(103–105)	ACE inhibitor	Inhibitor of Angiotensin-Converting Enzyme (ACE) (EC 3.4.15.1) (MEROPS ID: XM02-001)	ACE inhibitor	351.1680	351.3820
3	7513	PL	(106–107)	ACE inhibitor from Alaskan pollack skin	Inhibitor of Angiotensin-converting enzyme (EC 3.4.15.1) (MEROPS ID: XM02-001)	ACE inhibitor	228.1360	228.2770
4	7591	GF	(130–131)	ACE inhibitor	Inhibitor of Angiotensin-Converting Enzyme (ACE) (EC 3.4.15.1) (MEROPS ID: XM02-001)	ACE inhibitor	222.0890	222.2290
5	7599	GL	(183–184)	ACE inhibitor	Inhibitor of Angiotensin-converting enzyme (EC 3.4.15.1) (MEROPS ID: XM02-001)	ACE inhibitor	188.1050	188.2120
6	7693	KL	(21–22)	ACE inhibitor from wakame		ACE inhibitor	259.1780	259.3340
7	8219	TY	(23–24)	antioxidative peptide		antioxidative	282.1100	282.2750
8	8561	GL	(183–184)	dipeptidyl peptidase IV inhibitor (DPP IV inhibitor)	Inhibitor of Dipeptidyl Peptidase IV (EC 3.4.14.5) (MEROPS ID: S09.003)	dipeptidyl peptidase IV inhibitor	188.1050	188.2120
9	8638	PL	(106–107)	dipeptidyl peptidase IV inhibitor (DPP IV inhibitor)	Inhibitor of Dipeptidyl Peptidase IV (EC 3.4.14.5) (MEROPS ID: S09.003)	dipeptidyl peptidase IV inhibitor	228.1360	228.2770
10	8782	GF	(130–131)	dipeptidyl peptidase IV inhibitor (DPP IV inhibitor)	Inhibitor of Dipeptidyl Peptidase IV (EC 3.4.14.5) (MEROPS ID: S09.003)	dipeptidyl peptidase IV inhibitor	222.0890	222.2290
11	8886	RL	(138–139)	dipeptidyl peptidase IV inhibitor (DPP IV inhibitor)	Inhibitor of Dipeptidyl Peptidase IV (EC 3.4.14.5) (MEROPS ID: S09.003)	dipeptidyl peptidase IV inhibitor	287.1850	287.3480
12	8914	TY	(23–24)	dipeptidyl peptidase IV inhibitor (DPP IV inhibitor)	Inhibitor of Dipeptidyl Peptidase IV (EC 3.4.14.5) (MEROPS ID: S09.003)	dipeptidyl peptidase IV inhibitor	282.1100	282.2750
13	9071	IAY	(100–102)	ACE inhibitor	Inhibitor of Angiotensin-Converting Enzyme (ACE) (EC 3.4.15.1) (MEROPS ID: M02-001)	ACE inhibitor	365.1840	365.4090
14	9074	DF	(204–205)	ACE inhibitor	Inhibitor of Angiotensin-Converting Enzyme (ACE) (EC 3.4.15.1) (MEROPS ID: XM02-001)	ACE inhibitor	280.0949	280.2660

**Table A22:** *A_E_*, *DH_t_*, *W*, *B_E_* and *V* values from RubisCO of *Halophila stipulacea*. In silico enzymatic cleavage was carried out by cathepsin.

*DH_t_* (%)					
20.4878					
No.	Activity	*A_E_*	*W*	*B_E_*	*V*
1	ACE inhibitor	0.0388	0.0666	0.00082506965175237	0.030833564947006
2	antioxidative	0.0049	0.0673	0	0
3	dipeptidyl peptidase IV inhibitor	0.0243	0.0374	1.8563339357632E-6	0.0086054864513139

**Table A23:** Bioactive peptides from RubisCO of *Halophila stipulacea*. In silico enzymatic cleavage was carried out by using clostripain.

No.	Peptide ID	Sequence	Location	Name	Function	Activity	Monoisotopic Mass	Chemical Mass
1	8769	DR	(164–165)	dipeptidyl peptidase IV inhibitor (DPP IV inhibitor)	Inhibitor of Dipeptidyl Peptidase IV (EC 3.4.14.5) (MEROPS ID: S09.003)	dipeptidyl peptidase IV inhibitor	289.1279	289.2770

**Table A24:** *A_E_*, *DH_t_*, *W*, *B_E_* and *V* values from RubisCO of *Halophila stipulacea*. In silico enzymatic cleavage was carried out by clostripain.

*DH_t_* (%)					
5.3659					
No.	Activity	*A_E_*	*W*	*B_E_*	*V*
1	dipeptidyl peptidase IV inhibitor	0.0049	0.0075	0	0

**Table A25:** Bioactive peptides from RubisCO of *Halophila stipulacea*. In silico enzymatic cleavage was carried out by using chymase.

No.	Peptide ID	Sequence	Location	Name	Function	Activity	Monoisotopic Mass	Chemical Mass
1	3257	RL	(138–139)	beta-lactokinin	Inhibitor of Angiotensin-Converting Enzyme (ACE) (EC 3.4.15.1) (MEROPS ID: XM02-001)	ACE inhibitor	287.1850	287.3480
2	3546	VAY	(103–105)	ACE inhibitor	Inhibitor of Angiotensin-Converting Enzyme (ACE) (EC 3.4.15.1) (MEROPS ID: XM02-001)	ACE inhibitor	351.1680	351.3820
3	7513	PL	(106–107)	ACE inhibitor from Alaskan pollack skin	Inhibitor of Angiotensin-converting enzyme (EC 3.4.15.1) (MEROPS ID: XM02-001)	ACE inhibitor	228.1360	228.2770
4	7591	GF	(130–131)	ACE inhibitor	Inhibitor of Angiotensin-Converting Enzyme (ACE) (EC 3.4.15.1) (MEROPS ID: XM02-001)	ACE inhibitor	222.0890	222.2290
5	7599	GL	(183–184)	ACE inhibitor	Inhibitor of Angiotensin-converting enzyme (EC 3.4.15.1) (MEROPS ID: XM02-001)	ACE inhibitor	188.1050	188.2120
6	7693	KL	(21–22)	ACE inhibitor from wakame		ACE inhibitor	259.1780	259.3340
7	8219	TY	(23–24)	antioxidative peptide		antioxidative	282.1100	282.2750
8	8561	GL	(183–184)	dipeptidyl peptidase IV inhibitor (DPP IV inhibitor)	Inhibitor of Dipeptidyl Peptidase IV (EC 3.4.14.5) (MEROPS ID: S09.003)	dipeptidyl peptidase IV inhibitor	188.1050	188.2120
9	8638	PL	(106–107)	dipeptidyl peptidase IV inhibitor (DPP IV inhibitor)	Inhibitor of Dipeptidyl Peptidase IV (EC 3.4.14.5) (MEROPS ID: S09.003)	dipeptidyl peptidase IV inhibitor	228.1360	228.2770
10	8782	GF	(130–131)	dipeptidyl peptidase IV inhibitor (DPP IV inhibitor)	Inhibitor of Dipeptidyl Peptidase IV (EC 3.4.14.5) (MEROPS ID: S09.003)	dipeptidyl peptidase IV inhibitor	222.0890	222.2290
11	8886	RL	(138–139)	dipeptidyl peptidase IV inhibitor (DPP IV inhibitor)	Inhibitor of Dipeptidyl Peptidase IV (EC 3.4.14.5) (MEROPS ID: S09.003)	dipeptidyl peptidase IV inhibitor	287.1850	287.3480
12	8914	TY	(23–24)	dipeptidyl peptidase IV inhibitor (DPP IV inhibitor)	Inhibitor of Dipeptidyl Peptidase IV (EC 3.4.14.5) (MEROPS ID: S09.003)	dipeptidyl peptidase IV inhibitor	282.1100	282.2750
13	9071	IAY	(100–102)	ACE inhibitor	Inhibitor of Angiotensin-Converting Enzyme (ACE) (EC 3.4.15.1) (MEROPS ID: M02-001)	ACE inhibitor	365.1840	365.4090
14	9074	DF	(204–205)	ACE inhibitor	Inhibitor of Angiotensin-Converting Enzyme (ACE) (EC 3.4.15.1) (MEROPS ID: XM02-001)	ACE inhibitor	280.0949	280.2660

**Table A26:** *A_E_*, *DH_t_*, *W*, *B_E_* and *V* values from RubisCO of *Halophila stipulacea*. In silico enzymatic cleavage was carried out by chymase.

*DH_t_* (%)					
19.5122					
No.	Activity	*A_E_*	*W*	*B_E_*	*V*
1	ACE inhibitor	0.0388	0.0666	0.00082506965175237	0.030833564947006
2	antioxidative	0.0049	0.0673	0	0
3	dipeptidyl peptidase IV inhibitor	0.0243	0.0374	1.8563339357632E-6	0.0086054864513139

**Table A27:** Bioactive peptides from RubisCO of *Halophila stipulacea*. In silico enzymatic cleavage was carried out by using papain.

No.	Peptide ID	Sequence	Location	Name	Function	Activity	Monoisotopic Mass	Chemical Mass
1	3351	EEE	(95–97)	Stimulating vasoactive substance release		stimulating	405.1260	405.3480
2	3522	IPP	(144–146)	ACE inhibitor (from bovine b-CN)		ACE inhibitor	325.1880	325.3940
3	3538	VSP	(42–44)	ACE inhibitor	Inhibitor of Angiotensin-Converting Enzyme (ACE) (EC 3.4.15.1) (MEROPS ID: XM02-001)	ACE inhibitor	301.1520	301.3300
4	7513	PL	(172–173)	ACE inhibitor from Alaskan pollack skin	Inhibitor of Angiotensin-converting enzyme (EC 3.4.15.1) (MEROPS ID: XM02-001)	ACE inhibitor	228.1360	228.2770
5	7583	AF	(39–40)	ACE inhibitor	Inhibitor of Angiotensin-Converting Enzyme (ACE) (EC 3.4.15.1) (MEROPS ID: XM02-001)	ACE inhibitor	236.1050	236.2560
6	7594	VG	(11–12)	ACE inhibitor	Inhibitor of Angiotensin-Converting Enzyme (ACE) (EC 3.4.15.1) (MEROPS ID: XM02-001)	ACE inhibitor	174.0890	174.1850
7	7600	AG	(9–10)	ACE inhibitor	Inhibitor of Angiotensin-Converting Enzyme (ACE) (EC 3.4.15.1) (MEROPS ID: XM02-001)	ACE inhibitor	146.0580	146.1310
8	7600	AG	(15–16)	ACE inhibitor	Inhibitor of Angiotensin-Converting Enzyme (ACE) (EC 3.4.15.1) (MEROPS ID: XM02-001)	ACE inhibitor	146.0580	146.1310
9	7600	AG	(53–54)	ACE inhibitor	Inhibitor of Angiotensin-Converting Enzyme (ACE) (EC 3.4.15.1) (MEROPS ID: XM02-001)	ACE inhibitor	146.0580	146.1310
10	7600	AG	(93–94)	ACE inhibitor	Inhibitor of Angiotensin-Converting Enzyme (ACE) (EC 3.4.15.1) (MEROPS ID: XM02-001)	ACE inhibitor	146.0580	146.1310
11	7617	QG	(153–154)	ACE inhibitor		ACE inhibitor	203.0790	203.1830
12	7681	DG	(74–75)	ACE inhibitor from soy	Inhibitor of Angiotensin-converting enzyme (ACE) (EC 3.4.15.1) (MEROPS ID: XM02-001)	ACE inhibitor	190.0479	190.1410
13	7951	YYT	(24–26)	synthetic peptide		antioxidative	445.1730	445.4450
14	8559	AL	(133–134)	dipeptidyl peptidase IV inhibitor (DPP IV inhibitor)	Inhibitor of Dipeptidyl Peptidase IV (EC 3.4.14.5) (MEROPS ID: S09.003)	dipeptidyl peptidase IV inhibitor	202.1210	202.2390
15	8559	AL	(136–137)	dipeptidyl peptidase IV inhibitor (DPP IV inhibitor)	Inhibitor of Dipeptidyl Peptidase IV (EC 3.4.14.5) (MEROPS ID: S09.003)	dipeptidyl peptidase IV inhibitor	202.1210	202.2390
16	8560	SL	(78–79)	dipeptidyl peptidase IV inhibitor (DPP IV inhibitor)	Inhibitor of Dipeptidyl Peptidase IV (EC 3.4.14.5) (MEROPS ID: S09.003)	dipeptidyl peptidase IV inhibitor	218.1160	218.2400
17	8638	PL	(172–173)	dipeptidyl peptidase IV inhibitor (DPP IV inhibitor)	Inhibitor of Dipeptidyl Peptidase IV (EC 3.4.14.5) (MEROPS ID: S09.003)	dipeptidyl peptidase IV inhibitor	228.1360	228.2770
18	8685	WT	(68–69)	dipeptidyl peptidase IV inhibitor (DPP IV inhibitor)	Inhibitor of Dipeptidyl Peptidase IV (EC 3.4.14.5) (MEROPS ID: S09.003)	dipeptidyl peptidase IV inhibitor	305.1260	305.3180
19	8759	AF	(39–40)	dipeptidyl peptidase IV inhibitor (DPP IV inhibitor)	Inhibitor of Dipeptidyl Peptidase IV (EC 3.4.14.5) (MEROPS ID: S09.003)	dipeptidyl peptidase IV inhibitor	236.1050	236.2560
20	8760	AG	(9–10)	dipeptidyl peptidase IV inhibitor (DPP IV inhibitor)	Inhibitor of Dipeptidyl Peptidase IV (EC 3.4.14.5) (MEROPS ID: S09.003)	dipeptidyl peptidase IV inhibitor	146.0580	146.1310
21	8760	AG	(15–16)	dipeptidyl peptidase IV inhibitor (DPP IV inhibitor)	Inhibitor of Dipeptidyl Peptidase IV (EC 3.4.14.5) (MEROPS ID: S09.003)	dipeptidyl peptidase IV inhibitor	146.0580	146.1310
22	8760	AG	(53–54)	dipeptidyl peptidase IV inhibitor (DPP IV inhibitor)	Inhibitor of Dipeptidyl Peptidase IV (EC 3.4.14.5) (MEROPS ID: S09.003)	dipeptidyl peptidase IV inhibitor	146.0580	146.1310
23	8760	AG	(93–94)	dipeptidyl peptidase IV inhibitor (DPP IV inhibitor)	Inhibitor of Dipeptidyl Peptidase IV (EC 3.4.14.5) (MEROPS ID: S09.003)	dipeptidyl peptidase IV inhibitor	146.0580	146.1310
24	8764	AV	(56–57)	dipeptidyl peptidase IV inhibitor (DPP IV inhibitor)	Inhibitor of Dipeptidyl Peptidase IV (EC 3.4.14.5) (MEROPS ID: S09.003)	dipeptidyl peptidase IV inhibitor	188.1050	188.2120
25	8769	DR	(80–81)	dipeptidyl peptidase IV inhibitor (DPP IV inhibitor)	Inhibitor of Dipeptidyl Peptidase IV (EC 3.4.14.5) (MEROPS ID: S09.003)	dipeptidyl peptidase IV inhibitor	289.1279	289.2770
26	8769	DR	(164–165)	dipeptidyl peptidase IV inhibitor (DPP IV inhibitor)	Inhibitor of Dipeptidyl Peptidase IV (EC 3.4.14.5) (MEROPS ID: S09.003)	dipeptidyl peptidase IV inhibitor	289.1279	289.2770
27	8774	ET	(6–7)	dipeptidyl peptidase IV inhibitor (DPP IV inhibitor)	Inhibitor of Dipeptidyl Peptidase IV (EC 3.4.14.5) (MEROPS ID: S09.003)	dipeptidyl peptidase IV inhibitor	248.0890	248.2210
28	8871	QG	(153–154)	dipeptidyl peptidase IV inhibitor (DPP IV inhibitor)	Inhibitor of Dipeptidyl Peptidase IV (EC 3.4.14.5) (MEROPS ID: S09.003)	dipeptidyl peptidase IV inhibitor	203.0790	203.1830
29	8878	QT	(4–5)	dipeptidyl peptidase IV inhibitor (DPP IV inhibitor)	Inhibitor of Dipeptidyl Peptidase IV (EC 3.4.14.5) (MEROPS ID: S09.003)	dipeptidyl peptidase IV inhibitor	247.1050	247.2360
30	8918	VG	(11–12)	dipeptidyl peptidase IV inhibitor (DPP IV inhibitor)	Inhibitor of Dipeptidyl Peptidase IV (EC 3.4.14.5) (MEROPS ID: S09.003)	dipeptidyl peptidase IV inhibitor	174.0890	174.1850
31	8951	AV	(56–57)	ACE inhibitor	Inhibitor of Angiotensin-Converting Enzyme (ACE) (EC 3.4.15.1) (MEROPS ID: XM02-001)	ACE inhibitor	188.1050	188.2120
32	9074	DF	(204–205)	ACE inhibitor	Inhibitor of Angiotensin-Converting Enzyme (ACE) (EC 3.4.15.1) (MEROPS ID: XM02-001)	ACE inhibitor	280.0949	280.2660

**Table A28:** *A_E_*, *DH_t_*, *W*, *B_E_* and *V* values from RubisCO of *Halophila stipulacea*. In silico enzymatic cleavage was carried out by using papain.

*DH_t_* (%) 43.4146					
No.	Activity	*A_E_*	*W*	*B_E_*	*V*
1	stimulating	0.0049	0.1441	0	
2	ACE inhibitor	0.0631	0.1083	0.0019221967090735	0.071834149934202
3	antioxidative	0.0049	0.0673	0	0
4	dipeptidyl peptidase IV inhibitor	0.0825	0.1268	2.3003804384162E-5	0.10663971774841

**Table A29:** Bioactive peptides from RubisCO of *Halophila stipulacea*. In silico enzymatic cleavage was carried out by using ficin.

No.	Peptide ID	Sequence	Location	Name	Function	Activity	Monoisotopic Mass	Chemical Mass
1	2749	DY	(19–20)	ion flow regulating peptide	ion flow regulating peptide	regulating	296.0899	296.2590
2	3546	VAY	(103–105)	ACE inhibitor	Inhibitor of Angiotensin-Converting Enzyme (ACE) (EC 3.4.15.1) (MEROPS ID: XM02-001)	ACE inhibitor	351.1680	351.3820
3	7513	PL	(106–107)	ACE inhibitor from Alaskan pollack skin	Inhibitor of Angiotensin-converting enzyme (EC 3.4.15.1) (MEROPS ID: XM02-001)	ACE inhibitor	228.1360	228.2770
4	7513	PL	(172–173)	ACE inhibitor from Alaskan pollack skin	Inhibitor of Angiotensin-converting enzyme (EC 3.4.15.1) (MEROPS ID: XM02-001)	ACE inhibitor	228.1360	228.2770
5	7558	VK	(17–18)	ACE inhibitor from buckwheat		ACE inhibitor	245.1620	245.3070
6	7594	VG	(11–12)	ACE inhibitor	Inhibitor of Angiotensin-Converting Enzyme (ACE) (EC 3.4.15.1) (MEROPS ID: XM02-001)	ACE inhibitor	174.0890	174.1850
7	7600	AG	(9–10)	ACE inhibitor	Inhibitor of Angiotensin-Converting Enzyme (ACE) (EC 3.4.15.1) (MEROPS ID: XM02-001)	ACE inhibitor	146.0580	146.1310
8	7617	QG	(153–154)	ACE inhibitor		ACE inhibitor	203.0790	203.1830
9	7621	TG	(65–66)	ACE inhibitor		ACE inhibitor	176.0680	176.1570
10	7682	NY	(190–191)	ACE inhibitor from garlic		ACE inhibitor	295.1059	295.2740
11	7698	NK	(167–168)	ACE inhibitor from wakame		ACE inhibitor	260.1369	260.2780
12	8185	TF	(151–152)	ACE inhibitor	Inhibitor of Angiotensin-Converting Enzyme (ACE) (EC 3.4.15.1) (MEROPS ID: XM02-001)	ACE inhibitor	266.1150	266.2820
13	8219	TY	(23–24)	antioxidative peptide		antioxidative	282.1100	282.2750
14	8559	AL	(133–134)	dipeptidyl peptidase IV inhibitor (DPP IV inhibitor)	Inhibitor of Dipeptidyl Peptidase IV (EC 3.4.14.5) (MEROPS ID: S09.003)	dipeptidyl peptidase IV inhibitor	202.1210	202.2390
15	8559	AL	(136–137)	dipeptidyl peptidase IV inhibitor (DPP IV inhibitor)	Inhibitor of Dipeptidyl Peptidase IV (EC 3.4.14.5) (MEROPS ID: S09.003)	dipeptidyl peptidase IV inhibitor	202.1210	202.2390
16	8638	PL	(106–107)	dipeptidyl peptidase IV inhibitor (DPP IV inhibitor)	Inhibitor of Dipeptidyl Peptidase IV (EC 3.4.14.5) (MEROPS ID: S09.003)	dipeptidyl peptidase IV inhibitor	228.1360	228.2770
17	8638	PL	(172–173)	dipeptidyl peptidase IV inhibitor (DPP IV inhibitor)	Inhibitor of Dipeptidyl Peptidase IV (EC 3.4.14.5) (MEROPS ID: S09.003)	dipeptidyl peptidase IV inhibitor	228.1360	228.2770
18	8760	AG	(9–10)	dipeptidyl peptidase IV inhibitor (DPP IV inhibitor)	Inhibitor of Dipeptidyl Peptidase IV (EC 3.4.14.5) (MEROPS ID: S09.003)	dipeptidyl peptidase IV inhibitor	146.0580	146.1310
19	8769	DR	(80–81)	dipeptidyl peptidase IV inhibitor (DPP IV inhibitor)	Inhibitor of Dipeptidyl Peptidase IV (EC 3.4.14.5) (MEROPS ID: S09.003)	dipeptidyl peptidase IV inhibitor	289.1279	289.2770
20	8769	DR	(164–165)	dipeptidyl peptidase IV inhibitor (DPP IV inhibitor)	Inhibitor of Dipeptidyl Peptidase IV (EC 3.4.14.5) (MEROPS ID: S09.003)	dipeptidyl peptidase IV inhibitor	289.1279	289.2770
21	8853	NY	(190–191)	dipeptidyl peptidase IV inhibitor (DPP IV inhibitor)	Inhibitor of Dipeptidyl Peptidase IV (EC 3.4.14.5) (MEROPS ID: S09.003)	dipeptidyl peptidase IV inhibitor	295.1059	295.2740
22	8858	PK	(180–181)	dipeptidyl peptidase IV inhibitor (DPP IV inhibitor)	Inhibitor of Dipeptidyl Peptidase IV (EC 3.4.14.5) (MEROPS ID: S09.003)	dipeptidyl peptidase IV inhibitor	243.1460	243.2910
23	8871	QG	(153–154)	dipeptidyl peptidase IV inhibitor (DPP IV inhibitor)	Inhibitor of Dipeptidyl Peptidase IV (EC 3.4.14.5) (MEROPS ID: S09.003)	dipeptidyl peptidase IV inhibitor	203.0790	203.1830
24	8900	TF	(151–152)	dipeptidyl peptidase IV inhibitor (DPP IV inhibitor)	Inhibitor of Dipeptidyl Peptidase IV (EC 3.4.14.5) (MEROPS ID: S09.003)	dipeptidyl peptidase IV inhibitor	266.1150	266.2820
25	8901	TG	(65–66)	dipeptidyl peptidase IV inhibitor (DPP IV inhibitor)	Inhibitor of Dipeptidyl Peptidase IV (EC 3.4.14.5) (MEROPS ID: S09.003)	dipeptidyl peptidase IV inhibitor	176.0680	176.1570
26	8910	TS	(77–78)	dipeptidyl peptidase IV inhibitor (DPP IV inhibitor)	Inhibitor of Dipeptidyl Peptidase IV (EC 3.4.14.5) (MEROPS ID: S09.003)	dipeptidyl peptidase IV inhibitor	206.0790	206.1850
27	8910	TS	(120–121)	dipeptidyl peptidase IV inhibitor (DPP IV inhibitor)	Inhibitor of Dipeptidyl Peptidase IV (EC 3.4.14.5) (MEROPS ID: S09.003)	dipeptidyl peptidase IV inhibitor	206.0790	206.1850
28	8914	TY	(23–24)	dipeptidyl peptidase IV inhibitor (DPP IV inhibitor)	Inhibitor of Dipeptidyl Peptidase IV (EC 3.4.14.5) (MEROPS ID: S09.003)	dipeptidyl peptidase IV inhibitor	282.1100	282.2750
29	8918	VG	(11–12)	dipeptidyl peptidase IV inhibitor (DPP IV inhibitor)	Inhibitor of Dipeptidyl Peptidase IV (EC 3.4.14.5) (MEROPS ID: S09.003)	dipeptidyl peptidase IV inhibitor	174.0890	174.1850
30	8921	VK	(17–18)	dipeptidyl peptidase IV inhibitor (DPP IV inhibitor)	Inhibitor of Dipeptidyl Peptidase IV (EC 3.4.14.5) (MEROPS ID: S09.003)	dipeptidyl peptidase IV inhibitor	245.1620	245.3070
31	8926	VS	(42–43)	dipeptidyl peptidase IV inhibitor (DPP IV inhibitor)	Inhibitor of Dipeptidyl Peptidase IV (EC 3.4.14.5) (MEROPS ID: S09.003)	dipeptidyl peptidase IV inhibitor	204.1000	204.2130
32	9071	IAY	(100–102)	ACE inhibitor	Inhibitor of Angiotensin-Converting Enzyme (ACE) (EC 3.4.15.1) (MEROPS ID: M02-001)	ACE inhibitor	365.1840	365.4090
33	9072	DY	(19–20)	ACE inhibitor	Inhibitor of Angiotensin-Converting Enzyme (ACE) (EC 3.4.15.1) (MEROPS ID: XM02-001)	ACE inhibitor	296.0899	296.2590
34	9074	DF	(204–205)	ACE inhibitor	Inhibitor of Angiotensin-Converting Enzyme (ACE) (EC 3.4.15.1) (MEROPS ID: XM02-001)	ACE inhibitor	280.0949	280.2660

**Table A30:** *A_E_*, *DH_t_*, *W*, *B_E_* and *V* values from RubisCO of *Halophila stipulacea*. In silico enzymatic cleavage was carried out by using ficin.

*DH_t_* (%)					
44.3902					
No.	Activity	*A_E_*	*W*	*B_E_*	*V*
1	regulating	0.0049	0.3356	0	
2	ACE inhibitor	0.0680	0.1167	0.001585167723556	0.059239085878814
3	antioxidative	0.0049	0.0673	0	0
4	dipeptidyl peptidase IV inhibitor	0.0874	0.1344	1.1006017099609E-5	0.051021063187465

**Table A31:** Bioactive peptides from RubisCO of *Halophila stipulacea*. In silico enzymatic cleavage was carried out by using leukocyte elastase.

No.	Peptide ID	Sequence	Location	Name	Function	Activity	Monoisotopic Mass	Chemical Mass
1	3174	KA	(8–9)	dipeptidyl peptidase IV inhibitor (DPP IV inhibitor)	Inhibitor of Dipeptidyl Peptidase IV (EC 3.4.14.5) (MEROPS ID: S09.003)	dipeptidyl peptidase IV inhibitor	217.1310	217.2530
2	3257	RL	(138–139)	beta-lactokinin	Inhibitor of Angiotensin-Converting Enzyme (ACE) (EC 3.4.15.1) (MEROPS ID: XM02-001)	ACE inhibitor	287.1850	287.3480
3	4005	RA	(135–136)		Activation of ubiquitin-dependent proteolysis	activating ubiquitin-mediated proteolysis	245.1380	245.2670
4	7588	RA	(135–136)	ACE inhibitor	Inhibitor of Angiotensin-Converting Enzyme (ACE) (EC 3.4.15.1) (MEROPS ID: XM02-001)	ACE inhibitor	245.1380	245.2670
5	7598	GA	(54–55)	ACE inhibitor		ACE inhibitor	146.0580	146.1310
6	7599	GL	(183–184)	ACE inhibitor	Inhibitor of Angiotensin-converting enzyme (EC 3.4.15.1) (MEROPS ID: XM02-001)	ACE inhibitor	188.1050	188.2120
7	7608	GV	(10–11)	ACE inhibitor	Inhibitor of Angiotensin-Converting Enzyme (ACE) (EC 3.4.15.1) (MEROPS ID: XM02-001)	ACE inhibitor	174.0890	174.1850
8	7608	GV	(16–17)	ACE inhibitor	Inhibitor of Angiotensin-Converting Enzyme (ACE) (EC 3.4.15.1) (MEROPS ID: XM02-001)	ACE inhibitor	174.0890	174.1850
9	7612	GT	(66–67)	ACE inhibitor		ACE inhibitor	176.0680	176.1570
10	7743	KA	(8–9)	ACE inhibitor	Inhibitor of angiotensin-converting enzyme (EC 3.4.15.1) (MEROPS ID: XM02-001)	ACE inhibitor	217.1310	217.2530
11	7951	YYT	(24–26)	synthetic peptide		antioxidative	445.1730	445.4450
12	8524	GA	(54–55)	dipeptidyl peptidase IV inhibitor (DPP IV inhibitor)	Inhibitor of Dipeptidyl Peptidase IV (EC 3.4.14.5) (MEROPS ID: S09.003)	dipeptidyl peptidase IV inhibitor	146.0580	146.1310
13	8526	RA	(135–136)	dipeptidyl peptidase IV inhibitor (DPP IV inhibitor)	Inhibitor of Dipeptidyl Peptidase IV (EC 3.4.14.5) (MEROPS ID: S09.003)	dipeptidyl peptidase IV inhibitor	245.1380	245.2670
14	8561	GL	(183–184)	dipeptidyl peptidase IV inhibitor (DPP IV inhibitor)	Inhibitor of Dipeptidyl Peptidase IV (EC 3.4.14.5) (MEROPS ID: S09.003)	dipeptidyl peptidase IV inhibitor	188.1050	188.2120
15	8685	WT	(68–69)	dipeptidyl peptidase IV inhibitor (DPP IV inhibitor)	Inhibitor of Dipeptidyl Peptidase IV (EC 3.4.14.5) (MEROPS ID: S09.003)	dipeptidyl peptidase IV inhibitor	305.1260	305.3180
16	8685	WT	(72–73)	dipeptidyl peptidase IV inhibitor (DPP IV inhibitor)	Inhibitor of Dipeptidyl Peptidase IV (EC 3.4.14.5) (MEROPS ID: S09.003)	dipeptidyl peptidase IV inhibitor	305.1260	305.3180
17	8774	ET	(6–7)	dipeptidyl peptidase IV inhibitor (DPP IV inhibitor)	Inhibitor of Dipeptidyl Peptidase IV (EC 3.4.14.5) (MEROPS ID: S09.003)	dipeptidyl peptidase IV inhibitor	248.0890	248.2210
18	8786	GV	(10–11)	dipeptidyl peptidase IV inhibitor (DPP IV inhibitor)	Inhibitor of Dipeptidyl Peptidase IV (EC 3.4.14.5) (MEROPS ID: S09.003)	dipeptidyl peptidase IV inhibitor	174.0890	174.1850
19	8786	GV	(16–17)	dipeptidyl peptidase IV inhibitor (DPP IV inhibitor)	Inhibitor of Dipeptidyl Peptidase IV (EC 3.4.14.5) (MEROPS ID: S09.003)	dipeptidyl peptidase IV inhibitor	174.0890	174.1850
20	8816	KT	(150–151)	dipeptidyl peptidase IV inhibitor (DPP IV inhibitor)	Inhibitor of Dipeptidyl Peptidase IV (EC 3.4.14.5) (MEROPS ID: S09.003)	dipeptidyl peptidase IV inhibitor	247.1410	247.2790
21	8879	QV	(160–161)	dipeptidyl peptidase IV inhibitor (DPP IV inhibitor)	Inhibitor of Dipeptidyl Peptidase IV (EC 3.4.14.5) (MEROPS ID: S09.003)	dipeptidyl peptidase IV inhibitor	245.1260	245.2640
22	8884	RI	(143–144)	dipeptidyl peptidase IV inhibitor (DPP IV inhibitor)	Inhibitor of Dipeptidyl Peptidase IV (EC 3.4.14.5) (MEROPS ID: S09.003)	dipeptidyl peptidase IV inhibitor	287.1850	287.3480
23	8886	RL	(138–139)	dipeptidyl peptidase IV inhibitor (DPP IV inhibitor)	Inhibitor of Dipeptidyl Peptidase IV (EC 3.4.14.5) (MEROPS ID: S09.003)	dipeptidyl peptidase IV inhibitor	287.1850	287.3480
24	8945	YS	(148–149)	dipeptidyl peptidase IV inhibitor (DPP IV inhibitor)	Inhibitor of Dipeptidyl Peptidase IV (EC 3.4.14.5) (MEROPS ID: S09.003)	dipeptidyl peptidase IV inhibitor	268.0950	268.2500
25	8946	YV	(102–103)	dipeptidyl peptidase IV inhibitor (DPP IV inhibitor)	Inhibitor of Dipeptidyl Peptidase IV (EC 3.4.14.5) (MEROPS ID: S09.003)	dipeptidyl peptidase IV inhibitor	280.1310	280.3030
26	9056	DGL	(74–76)	ACE inhibitor	Inhibitor of Angiotensin-Converting Enzyme (ACE) (EC 3.4.15.1) (MEROPS ID: XM02-001)	ACE inhibitor	303.1319	303.3010
27	9077	YV	(102–103)	ACE inhibitor	Inhibitor of Angiotensin-Converting Enzyme (ACE) (EC 3.4.15.1) (MEROPS ID: XM02-001)	ACE inhibitor	280.1310	280.3030

**Table A32:** *A_E_*, *DH_t_*, *W*, *B_E_* and *V* values from RubisCO of *Halophila stipulacea*. In silico enzymatic cleavage was carried out by using leukocyte elastase.

*DH_t_* (%)					
38.5366					
No.	Activity	*A_E_*	*W*	*B_E_*	*V*
1	dipeptidyl peptidase IV inhibitor	0.0728	0.1119	2.2768987034696E-5	0.10555116493987
2	ACE inhibitor	0.0485	0.0833	0.0024508146173148	0.091589057379035
3	activating ubiquitin-mediated proteolysis	0.0049	0.3356	0	
4	antioxidative	0.0049	0.0673	0	0

**Table A33:** Bioactive peptides from RubisCO of *Halophila stipulacea*. In silico enzymatic cleavage was carried out by using metridin.

No.	Peptide ID	Sequence	Location	Name	Function	Activity	Monoisotopic Mass	Chemical Mass
1	3257	RL	(138–139)	beta-lactokinin	Inhibitor of Angiotensin-Converting Enzyme (ACE) (EC 3.4.15.1) (MEROPS ID: XM02-001)	ACE inhibitor	287.1850	287.3480
2	3546	VAY	(103–105)	ACE inhibitor	Inhibitor of Angiotensin-Converting Enzyme (ACE) (EC 3.4.15.1) (MEROPS ID: XM02-001)	ACE inhibitor	351.1680	351.3820
3	7513	PL	(106–107)	ACE inhibitor from Alaskan pollack skin	Inhibitor of Angiotensin-converting enzyme (EC 3.4.15.1) (MEROPS ID: XM02-001)	ACE inhibitor	228.1360	228.2770
4	7591	GF	(130–131)	ACE inhibitor	Inhibitor of Angiotensin-Converting Enzyme (ACE) (EC 3.4.15.1) (MEROPS ID: XM02-001)	ACE inhibitor	222.0890	222.2290
5	7599	GL	(183–184)	ACE inhibitor	Inhibitor of Angiotensin-converting enzyme (EC 3.4.15.1) (MEROPS ID: XM02-001)	ACE inhibitor	188.1050	188.2120
6	7693	KL	(21–22)	ACE inhibitor from wakame		ACE inhibitor	259.1780	259.3340
7	8219	TY	(23–24)	antioxidative peptide		antioxidative	282.1100	282.2750
8	8561	GL	(183–184)	dipeptidyl peptidase IV inhibitor (DPP IV inhibitor)	Inhibitor of Dipeptidyl Peptidase IV (EC 3.4.14.5) (MEROPS ID: S09.003)	dipeptidyl peptidase IV inhibitor	188.1050	188.2120
9	8638	PL	(106–107)	dipeptidyl peptidase IV inhibitor (DPP IV inhibitor)	Inhibitor of Dipeptidyl Peptidase IV (EC 3.4.14.5) (MEROPS ID: S09.003)	dipeptidyl peptidase IV inhibitor	228.1360	228.2770
10	8782	GF	(130–131)	dipeptidyl peptidase IV inhibitor (DPP IV inhibitor)	Inhibitor of Dipeptidyl Peptidase IV (EC 3.4.14.5) (MEROPS ID: S09.003)	dipeptidyl peptidase IV inhibitor	222.0890	222.2290
11	8886	RL	(138–139)	dipeptidyl peptidase IV inhibitor (DPP IV inhibitor)	Inhibitor of Dipeptidyl Peptidase IV (EC 3.4.14.5) (MEROPS ID: S09.003)	dipeptidyl peptidase IV inhibitor	287.1850	287.3480
12	8914	TY	(23–24)	dipeptidyl peptidase IV inhibitor (DPP IV inhibitor)	Inhibitor of Dipeptidyl Peptidase IV (EC 3.4.14.5) (MEROPS ID: S09.003)	dipeptidyl peptidase IV inhibitor	282.1100	282.2750
13	9071	IAY	(100–102)	ACE inhibitor	Inhibitor of Angiotensin-Converting Enzyme (ACE) (EC 3.4.15.1) (MEROPS ID: M02-001)	ACE inhibitor	365.1840	365.4090
14	9074	DF	(204–205)	ACE inhibitor	Inhibitor of Angiotensin-Converting Enzyme (ACE) (EC 3.4.15.1) (MEROPS ID: XM02-001)	ACE inhibitor	280.0949	280.2660

**Table A34:** *A_E_*, *DH_t_*, *W*, *B_E_* and *V* values from RubisCO of *Halophila stipulacea*. In silico enzymatic cleavage was carried out by using metridin.

*DH_t_* (%)					
19.5122					
No.	Activity	*A_E_*	*W*	*B_E_*	*V*
1	ACE inhibitor	0.0388	0.0666	0.00082506965175237	0.030833564947006
2	antioxidative	0.0049	0.0673	0	0
3	dipeptidyl peptidase IV inhibitor	0.0243	0.0374	1.8563339357632E-6	0.0086054864513139

**Table A35:** Bioactive peptides from RubisCO of *Halophila stipulacea*. In silico enzymatic cleavage was carried out by using pancreatic elastase II.

No.	Peptide ID	Sequence	Location	Name	Function	Activity	Monoisotopic Mass	Chemical Mass
1	3257	RL	(138–139)	beta-lactokinin	Inhibitor of Angiotensin-Converting Enzyme (ACE) (EC 3.4.15.1) (MEROPS ID: XM02-001)	ACE inhibitor	287.1850	287.3480
2	7591	GF	(130–131)	ACE inhibitor	Inhibitor of Angiotensin-Converting Enzyme (ACE) (EC 3.4.15.1) (MEROPS ID: XM02-001)	ACE inhibitor	222.0890	222.2290
3	7599	GL	(183–184)	ACE inhibitor	Inhibitor of Angiotensin-converting enzyme (EC 3.4.15.1) (MEROPS ID: XM02-001)	ACE inhibitor	188.1050	188.2120
4	8561	GL	(183–184)	dipeptidyl peptidase IV inhibitor (DPP IV inhibitor)	Inhibitor of Dipeptidyl Peptidase IV (EC 3.4.14.5) (MEROPS ID: S09.003)	dipeptidyl peptidase IV inhibitor	188.1050	188.2120
5	8782	GF	(130–131)	dipeptidyl peptidase IV inhibitor (DPP IV inhibitor)	Inhibitor of Dipeptidyl Peptidase IV (EC 3.4.14.5) (MEROPS ID: S09.003)	dipeptidyl peptidase IV inhibitor	222.0890	222.2290
6	8886	RL	(138–139)	dipeptidyl peptidase IV inhibitor (DPP IV inhibitor)	Inhibitor of Dipeptidyl Peptidase IV (EC 3.4.14.5) (MEROPS ID: S09.003)	dipeptidyl peptidase IV inhibitor	287.1850	287.3480
7	9074	DF	(204–205)	ACE inhibitor	Inhibitor of Angiotensin-Converting Enzyme (ACE) (EC 3.4.15.1) (MEROPS ID: XM02-001)	ACE inhibitor	280.0949	280.2660

**Table A36:** *A_E_*, *DH_t_*, *W*, *B_E_* and *V* values from RubisCO of *Halophila stipulacea*. In silico enzymatic cleavage was carried out by using pancreatic elastase II.

*DH_t_* (%)					
13.1707					
No.	Activity	*A_E_*	*W*	*B_E_*	*V*
1	ACE inhibitor	0.0194	0.0333	2.5006640432763E-5	0.00093451973448837
2	dipeptidyl peptidase IV inhibitor	0.0146	0.0224	1.8563339357632E-6	0.0086054864513139

**Table A37:** Bioactive peptides from RubisCO of *Halophila stipulacea*. In silico enzymatic cleavage was carried out by using stem bromelain.

No.	Peptide ID	Sequence	Location	Name	Function	Activity	Monoisotopic Mass	Chemical Mass
1	3174	KA	(8–9)	dipeptidyl peptidase IV inhibitor (DPP IV inhibitor)	Inhibitor of Dipeptidyl Peptidase IV (EC 3.4.14.5) (MEROPS ID: S09.003)	dipeptidyl peptidase IV inhibitor	217.1310	217.2530
2	3174	KA	(132–133)	dipeptidyl peptidase IV inhibitor (DPP IV inhibitor)	Inhibitor of Dipeptidyl Peptidase IV (EC 3.4.14.5) (MEROPS ID: S09.003)	dipeptidyl peptidase IV inhibitor	217.1310	217.2530
3	7513	PL	(172–173)	ACE inhibitor from Alaskan pollack skin	Inhibitor of Angiotensin-converting enzyme (EC 3.4.15.1) (MEROPS ID: XM02-001)	ACE inhibitor	228.1360	228.2770
4	7617	QG	(153–154)	ACE inhibitor		ACE inhibitor	203.0790	203.1830
5	7681	DG	(74–75)	ACE inhibitor from soy	Inhibitor of Angiotensin-converting enzyme (ACE) (EC 3.4.15.1) (MEROPS ID: XM02-001)	ACE inhibitor	190.0479	190.1410
6	7743	KA	(8–9)	ACE inhibitor	Inhibitor of angiotensin-converting enzyme (EC 3.4.15.1) (MEROPS ID: XM02-001)	ACE inhibitor	217.1310	217.2530
7	7743	KA	(132–133)	ACE inhibitor	Inhibitor of angiotensin-converting enzyme (EC 3.4.15.1) (MEROPS ID: XM02-001)	ACE inhibitor	217.1310	217.2530
8	7951	YYT	(24–26)	synthetic peptide		antioxidative	445.1730	445.4450
9	8638	PL	(172–173)	dipeptidyl peptidase IV inhibitor (DPP IV inhibitor)	Inhibitor of Dipeptidyl Peptidase IV (EC 3.4.14.5) (MEROPS ID: S09.003)	dipeptidyl peptidase IV inhibitor	228.1360	228.2770
10	8685	WT	(68–69)	dipeptidyl peptidase IV inhibitor (DPP IV inhibitor)	Inhibitor of Dipeptidyl Peptidase IV (EC 3.4.14.5) (MEROPS ID: S09.003)	dipeptidyl peptidase IV inhibitor	305.1260	305.3180
11	8685	WT	(72–73)	dipeptidyl peptidase IV inhibitor (DPP IV inhibitor)	Inhibitor of Dipeptidyl Peptidase IV (EC 3.4.14.5) (MEROPS ID: S09.003)	dipeptidyl peptidase IV inhibitor	305.1260	305.3180
12	8769	DR	(80–81)	dipeptidyl peptidase IV inhibitor (DPP IV inhibitor)	Inhibitor of Dipeptidyl Peptidase IV (EC 3.4.14.5) (MEROPS ID: S09.003)	dipeptidyl peptidase IV inhibitor	289.1279	289.2770
13	8769	DR	(164–165)	dipeptidyl peptidase IV inhibitor (DPP IV inhibitor)	Inhibitor of Dipeptidyl Peptidase IV (EC 3.4.14.5) (MEROPS ID: S09.003)	dipeptidyl peptidase IV inhibitor	289.1279	289.2770
14	8774	ET	(6–7)	dipeptidyl peptidase IV inhibitor (DPP IV inhibitor)	Inhibitor of Dipeptidyl Peptidase IV (EC 3.4.14.5) (MEROPS ID: S09.003)	dipeptidyl peptidase IV inhibitor	248.0890	248.2210
15	8816	KT	(150–151)	dipeptidyl peptidase IV inhibitor (DPP IV inhibitor)	Inhibitor of Dipeptidyl Peptidase IV (EC 3.4.14.5) (MEROPS ID: S09.003)	dipeptidyl peptidase IV inhibitor	247.1410	247.2790
16	8851	NV	(127–128)	dipeptidyl peptidase IV inhibitor (DPP IV inhibitor)	Inhibitor of Dipeptidyl Peptidase IV (EC 3.4.14.5) (MEROPS ID: S09.003)	dipeptidyl peptidase IV inhibitor	231.1109	231.2370
17	8867	QA	(14–15)	dipeptidyl peptidase IV inhibitor (DPP IV inhibitor)	Inhibitor of Dipeptidyl Peptidase IV (EC 3.4.14.5) (MEROPS ID: S09.003)	dipeptidyl peptidase IV inhibitor	217.0950	217.2100
18	8871	QG	(153–154)	dipeptidyl peptidase IV inhibitor (DPP IV inhibitor)	Inhibitor of Dipeptidyl Peptidase IV (EC 3.4.14.5) (MEROPS ID: S09.003)	dipeptidyl peptidase IV inhibitor	203.0790	203.1830
19	8945	YS	(148–149)	dipeptidyl peptidase IV inhibitor (DPP IV inhibitor)	Inhibitor of Dipeptidyl Peptidase IV (EC 3.4.14.5) (MEROPS ID: S09.003)	dipeptidyl peptidase IV inhibitor	268.0950	268.2500
20	8946	YV	(102–103)	dipeptidyl peptidase IV inhibitor (DPP IV inhibitor)	Inhibitor of Dipeptidyl Peptidase IV (EC 3.4.14.5) (MEROPS ID: S09.003)	dipeptidyl peptidase IV inhibitor	280.1310	280.3030
21	9074	DF	(204–205)	ACE inhibitor	Inhibitor of Angiotensin-Converting Enzyme (ACE) (EC 3.4.15.1) (MEROPS ID: XM02-001)	ACE inhibitor	280.0949	280.2660
22	9077	YV	(102–103)	ACE inhibitor	Inhibitor of Angiotensin-Converting Enzyme (ACE) (EC 3.4.15.1) (MEROPS ID: XM02-001)	ACE inhibitor	280.1310	280.3030

**Table A38:** *A_E_*, *DH_t_*, *W*, *B_E_* and *V* values from RubisCO of *Halophila stipulacea*. In silico enzymatic cleavage was carried out by using stem bromelain.

*DH_t_* (%)					
54.1463					
No.	Activity	*A_E_*	*W*	*B_E_*	*V*
1	dipeptidyl peptidase IV inhibitor	0.0680	0.1045	2.1686874619195E-5	0.10053477023254
2	ACE inhibitor	0.0340	0.0584	0.00073973077367093	0.027644377422971
3	antioxidative	0.0049	0.0673	0	0

**Table A39:** Bioactive peptides from RubisCO of *Halophila stipulacea*. In silico enzymatic cleavage was carried out by using oligopeptidase B.

No.	Peptide ID	Sequence	Location	Name	Function	Activity	Monoisotopic Mass	Chemical Mass
1	7603	GR	(84–85)	ACE inhibitor	Inhibitor of Angiotensin-Converting Enzyme (ACE) (EC 3.4.15.1) (MEROPS ID: XM02-001)	ACE inhibitor	231.1220	231.2400
2	7697	YK	(82–83)	ACE inhibitor from wakame		ACE inhibitor	309.1570	309.3440
3	8769	DR	(164–165)	dipeptidyl peptidase IV inhibitor (DPP IV inhibitor)	Inhibitor of Dipeptidyl Peptidase IV (EC 3.4.14.5) (MEROPS ID: S09.003)	dipeptidyl peptidase IV inhibitor	289.1279	289.2770
4	8858	PK	(180–181)	dipeptidyl peptidase IV inhibitor (DPP IV inhibitor)	Inhibitor of Dipeptidyl Peptidase IV (EC 3.4.14.5) (MEROPS ID: S09.003)	dipeptidyl peptidase IV inhibitor	243.1460	243.2910
5	8939	YK	(82–83)	dipeptidyl peptidase IV inhibitor (DPP IV inhibitor)	Inhibitor of Dipeptidyl Peptidase IV (EC 3.4.14.5) (MEROPS ID: S09.003)	dipeptidyl peptidase IV inhibitor	309.1570	309.3440

**Table A40:** *A_E_*, *DH_t_*, *W*, *B_E_* and *V* values from RubisCO of *Halophila stipulacea*. In silico enzymatic cleavage was carried out by using oligopeptidase B.

*DH_t_* (%)					
10.7317					
No.	Activity	*A_E_*	*W*	*B_E_*	*V*
1	ACE inhibitor	0.0097	0.0167	9.4749721470635E-6	0.00035408788633428
2	dipeptidyl peptidase IV inhibitor	0.0146	0.0224	0	0

**Table A41:** Bioactive peptides from RubisCO of *Halophila stipulacea*. In silico enzymatic cleavage was carried out by using calpain 2.

No.	Peptide ID	Sequence	Location	Name	Function	Activity	Monoisotopic Mass	Chemical Mass
1	2754	PG	(46–47)	peptide regulating the stomach mucosal membrane activity	regulating the stomach mucosal membrane activity	regulating	172.0730	172.1690
2	2882	YG	(169–170)	Immunostimulating peptide	Enhancing protein biosynthesis in lymphocytes	immunomodulating	238.0840	238.2220
3	3285	PG	(46–47)	Antithrombotic peptide	Antithrombotic	antithrombotic	172.0730	172.1690
4	3460	PG	(46–47)	Prolyl endopeptidase inhibitor	Inhibitor of Prolyl Endopeptidase (PEP) (EC 3.4.21.26) (MEROPS ID: S09.001)	antiamnestic	172.0730	172.1690
5	3522	IPP	(144–146)	ACE inhibitor (from bovine b-CN)		ACE inhibitor	325.1880	325.3940
6	3553	YG	(169–170)	ACE inhibitor	Inhibitor of Angiotensin-Converting Enzyme (ACE) (EC 3.4.15.1) (MEROPS ID: XM02-001)	ACE inhibitor	238.0840	238.2220
7	3563	AY	(147–148)	ACE inhibitor	Inhibitor of Angiotensin-Converting Enzyme (ACE) (EC 3.4.15.1) (MEROPS ID: XM02-001)	ACE inhibitor	252.1000	252.2490
8	7513	PL	(172–173)	ACE inhibitor from Alaskan pollack skin	Inhibitor of Angiotensin-converting enzyme (EC 3.4.15.1) (MEROPS ID: XM02-001)	ACE inhibitor	228.1360	228.2770
9	7558	VK	(17–18)	ACE inhibitor from buckwheat		ACE inhibitor	245.1620	245.3070
10	7594	VG	(11–12)	ACE inhibitor	Inhibitor of Angiotensin-Converting Enzyme (ACE) (EC 3.4.15.1) (MEROPS ID: XM02-001)	ACE inhibitor	174.0890	174.1850
11	7600	AG	(9–10)	ACE inhibitor	Inhibitor of Angiotensin-Converting Enzyme (ACE) (EC 3.4.15.1) (MEROPS ID: XM02-001)	ACE inhibitor	146.0580	146.1310
12	7600	AG	(15–16)	ACE inhibitor	Inhibitor of Angiotensin-Converting Enzyme (ACE) (EC 3.4.15.1) (MEROPS ID: XM02-001)	ACE inhibitor	146.0580	146.1310
13	7600	AG	(53–54)	ACE inhibitor	Inhibitor of Angiotensin-Converting Enzyme (ACE) (EC 3.4.15.1) (MEROPS ID: XM02-001)	ACE inhibitor	146.0580	146.1310
14	7600	AG	(93–94)	ACE inhibitor	Inhibitor of Angiotensin-Converting Enzyme (ACE) (EC 3.4.15.1) (MEROPS ID: XM02-001)	ACE inhibitor	146.0580	146.1310
15	7625	PG	(46–47)	ACE inhibitor	Inhibitor of Angiotensin-Converting Enzyme (ACE) (EC 3.4.15.1) (MEROPS ID: M02-001)	ACE inhibitor	172.0730	172.1690
16	7681	DG	(74–75)	ACE inhibitor from soy	Inhibitor of Angiotensin-converting enzyme (ACE) (EC 3.4.15.1) (MEROPS ID: XM02-001)	ACE inhibitor	190.0479	190.1410
17	7697	YK	(82–83)	ACE inhibitor from wakame		ACE inhibitor	309.1570	309.3440
18	7698	NK	(167–168)	ACE inhibitor from wakame		ACE inhibitor	260.1369	260.2780
19	7866	AY	(147–148)	peptide from Okara protein	Peptide obtained by hydrolysis of Okara protein by use of enzymatic preparation Protease N.	antioxidative	252.1000	252.2490
20	7951	YYT	(24–26)	synthetic peptide		antioxidative	445.1730	445.4450
21	8559	AL	(133–134)	dipeptidyl peptidase IV inhibitor (DPP IV inhibitor)	Inhibitor of Dipeptidyl Peptidase IV (EC 3.4.14.5) (MEROPS ID: S09.003)	dipeptidyl peptidase IV inhibitor	202.1210	202.2390
22	8559	AL	(136–137)	dipeptidyl peptidase IV inhibitor (DPP IV inhibitor)	Inhibitor of Dipeptidyl Peptidase IV (EC 3.4.14.5) (MEROPS ID: S09.003)	dipeptidyl peptidase IV inhibitor	202.1210	202.2390
23	8560	SL	(78–79)	dipeptidyl peptidase IV inhibitor (DPP IV inhibitor)	Inhibitor of Dipeptidyl Peptidase IV (EC 3.4.14.5) (MEROPS ID: S09.003)	dipeptidyl peptidase IV inhibitor	218.1160	218.2400
24	8638	PL	(172–173)	dipeptidyl peptidase IV inhibitor (DPP IV inhibitor)	Inhibitor of Dipeptidyl Peptidase IV (EC 3.4.14.5) (MEROPS ID: S09.003)	dipeptidyl peptidase IV inhibitor	228.1360	228.2770
25	8685	WT	(68–69)	dipeptidyl peptidase IV inhibitor (DPP IV inhibitor)	Inhibitor of Dipeptidyl Peptidase IV (EC 3.4.14.5) (MEROPS ID: S09.003)	dipeptidyl peptidase IV inhibitor	305.1260	305.3180
26	8760	AG	(9–10)	dipeptidyl peptidase IV inhibitor (DPP IV inhibitor)	Inhibitor of Dipeptidyl Peptidase IV (EC 3.4.14.5) (MEROPS ID: S09.003)	dipeptidyl peptidase IV inhibitor	146.0580	146.1310
27	8760	AG	(15–16)	dipeptidyl peptidase IV inhibitor (DPP IV inhibitor)	Inhibitor of Dipeptidyl Peptidase IV (EC 3.4.14.5) (MEROPS ID: S09.003)	dipeptidyl peptidase IV inhibitor	146.0580	146.1310
28	8760	AG	(53–54)	dipeptidyl peptidase IV inhibitor (DPP IV inhibitor)	Inhibitor of Dipeptidyl Peptidase IV (EC 3.4.14.5) (MEROPS ID: S09.003)	dipeptidyl peptidase IV inhibitor	146.0580	146.1310
29	8760	AG	(93–94)	dipeptidyl peptidase IV inhibitor (DPP IV inhibitor)	Inhibitor of Dipeptidyl Peptidase IV (EC 3.4.14.5) (MEROPS ID: S09.003)	dipeptidyl peptidase IV inhibitor	146.0580	146.1310
30	8764	AV	(56–57)	dipeptidyl peptidase IV inhibitor (DPP IV inhibitor)	Inhibitor of Dipeptidyl Peptidase IV (EC 3.4.14.5) (MEROPS ID: S09.003)	dipeptidyl peptidase IV inhibitor	188.1050	188.2120
31	8765	AY	(147–148)	dipeptidyl peptidase IV inhibitor (DPP IV inhibitor)	Inhibitor of Dipeptidyl Peptidase IV (EC 3.4.14.5) (MEROPS ID: S09.003)	dipeptidyl peptidase IV inhibitor	252.1000	252.2490
32	8769	DR	(80–81)	dipeptidyl peptidase IV inhibitor (DPP IV inhibitor)	Inhibitor of Dipeptidyl Peptidase IV (EC 3.4.14.5) (MEROPS ID: S09.003)	dipeptidyl peptidase IV inhibitor	289.1279	289.2770
33	8769	DR	(164–165)	dipeptidyl peptidase IV inhibitor (DPP IV inhibitor)	Inhibitor of Dipeptidyl Peptidase IV (EC 3.4.14.5) (MEROPS ID: S09.003)	dipeptidyl peptidase IV inhibitor	289.1279	289.2770
34	8774	ET	(6–7)	dipeptidyl peptidase IV inhibitor (DPP IV inhibitor)	Inhibitor of Dipeptidyl Peptidase IV (EC 3.4.14.5) (MEROPS ID: S09.003)	dipeptidyl peptidase IV inhibitor	248.0890	248.2210
35	8779	FQ	(13–14)	dipeptidyl peptidase IV inhibitor (DPP IV inhibitor)	Inhibitor of Dipeptidyl Peptidase IV (EC 3.4.14.5) (MEROPS ID: S09.003)	dipeptidyl peptidase IV inhibitor	293.1260	293.3080
36	8779	FQ	(152–153)	dipeptidyl peptidase IV inhibitor (DPP IV inhibitor)	Inhibitor of Dipeptidyl Peptidase IV (EC 3.4.14.5) (MEROPS ID: S09.003)	dipeptidyl peptidase IV inhibitor	293.1260	293.3080
37	8805	IQ	(159–160)	dipeptidyl peptidase IV inhibitor (DPP IV inhibitor)	Inhibitor of Dipeptidyl Peptidase IV (EC 3.4.14.5) (MEROPS ID: S09.003)	dipeptidyl peptidase IV inhibitor	259.1420	259.2910
38	8855	PG	(46–47)	dipeptidyl peptidase IV inhibitor (DPP IV inhibitor)	Inhibitor of Dipeptidyl Peptidase IV (EC 3.4.14.5) (MEROPS ID: S09.003)	dipeptidyl peptidase IV inhibitor	172.0730	172.1690
39	8858	PK	(180–181)	dipeptidyl peptidase IV inhibitor (DPP IV inhibitor)	Inhibitor of Dipeptidyl Peptidase IV (EC 3.4.14.5) (MEROPS ID: S09.003)	dipeptidyl peptidase IV inhibitor	243.1460	243.2910
40	8894	SK	(149–150)	dipeptidyl peptidase IV inhibitor (DPP IV inhibitor)	Inhibitor of Dipeptidyl Peptidase IV (EC 3.4.14.5) (MEROPS ID: S09.003)	dipeptidyl peptidase IV inhibitor	233.1260	233.2540
41	8918	VG	(11–12)	dipeptidyl peptidase IV inhibitor (DPP IV inhibitor)	Inhibitor of Dipeptidyl Peptidase IV (EC 3.4.14.5) (MEROPS ID: S09.003)	dipeptidyl peptidase IV inhibitor	174.0890	174.1850
42	8921	VK	(17–18)	dipeptidyl peptidase IV inhibitor (DPP IV inhibitor)	Inhibitor of Dipeptidyl Peptidase IV (EC 3.4.14.5) (MEROPS ID: S09.003)	dipeptidyl peptidase IV inhibitor	245.1620	245.3070
43	8936	YG	(169–170)	dipeptidyl peptidase IV inhibitor (DPP IV inhibitor)	Inhibitor of Dipeptidyl Peptidase IV (EC 3.4.14.5) (MEROPS ID: S09.003)	dipeptidyl peptidase IV inhibitor	238.0840	238.2220
44	8938	YI	(99–100)	dipeptidyl peptidase IV inhibitor (DPP IV inhibitor)	Inhibitor of Dipeptidyl Peptidase IV (EC 3.4.14.5) (MEROPS ID: S09.003)	dipeptidyl peptidase IV inhibitor	294.1470	294.3300
45	8939	YK	(82–83)	dipeptidyl peptidase IV inhibitor (DPP IV inhibitor)	Inhibitor of Dipeptidyl Peptidase IV (EC 3.4.14.5) (MEROPS ID: S09.003)	dipeptidyl peptidase IV inhibitor	309.1570	309.3440
46	8951	AV	(56–57)	ACE inhibitor	Inhibitor of Angiotensin-Converting Enzyme (ACE) (EC 3.4.15.1) (MEROPS ID: XM02-001)	ACE inhibitor	188.1050	188.2120
47	9076	FQ	(13–14)	ACE inhibitor	Inhibitor of Angiotensin-Converting Enzyme (ACE) (EC 3.4.15.1) (MEROPS ID: XM02-001)	ACE inhibitor	293.1260	293.3080
48	9076	FQ	(152–153)	ACE inhibitor	Inhibitor of Angiotensin-Converting Enzyme (ACE) (EC 3.4.15.1) (MEROPS ID: XM02-001)	ACE inhibitor	293.1260	293.3080
49	9184	ST	(64–65)	ACE inhibitor	Inhibitor of Angiotensin-Converting Enzyme (ACE) (EC 3.4.15.1) (MEROPS ID: M02-001)	ACE inhibitor	206.0790	206.1850

**Table A42:** *A_E_*, *DH_t_*, *W*, *B_E_* and *V* values from RubisCO of *Halophila stipulacea*. In silico enzymatic cleavage was carried out by using calpain 2.

*DH_t_* (%)					
47.3171					
No.	Activity	*A_E_*	*W*	*B_E_*	*V*
1	regulating	0.0049	0.3356	0	
2	immunomodulating	0.0049	0.5052	0	
3	antithrombotic	0.0049	0.5052	0	
4	antiamnestic	0.0049	0.5052	0	
5	ACE inhibitor	0.0874	0.1500	0.0034373644220009	0.12845727500362
6	antioxidative	0.0097	0.1332	0	0
7	dipeptidyl peptidase IV inhibitor	0.1214	0.1866	2.3003804384162E-5	0.10663971774841

**Table A43:** Bioactive peptides from RubisCO of *Halophila stipulacea*. In silico enzymatic cleavage was carried out by using glycyl endopeptidase.

No.	Peptide ID	Sequence	Location	Name	Function	Activity	Monoisotopic Mass	Chemical Mass
1	7594	VG	(11–12)	ACE inhibitor	Inhibitor of Angiotensin-Converting Enzyme (ACE) (EC 3.4.15.1) (MEROPS ID: XM02-001)	ACE inhibitor	174.0890	174.1850
2	8918	VG	(11–12)	dipeptidyl peptidase IV inhibitor (DPP IV inhibitor)	Inhibitor of Dipeptidyl Peptidase IV (EC 3.4.14.5) (MEROPS ID: S09.003)	dipeptidyl peptidase IV inhibitor	174.0890	174.1850

**Table A44:** *A_E_*, *DH_t_*, *W*, *B_E_* and *V* values from RubisCO of *Halophila stipulacea*. In silico enzymatic cleavage was carried out by using glycyl endopeptidase.

*DH_t_* (%)					
9.7561					
No.	Activity	*A_E_*	*W*	*B_E_*	*V*
1	ACE inhibitor	0.0049	0.0084	4.4130626654898E-6	0.00016491996042102
2	dipeptidyl peptidase IV inhibitor	0.0049	0.0075	0	0

**Table A45:** Bioactive peptides from RubisCO of *Halophila stipulacea*. In silico enzymatic cleavage was carried out by using oligopeptidase F.

No.	Peptide ID	Sequence	Location	Name	Function	Activity	Monoisotopic Mass	Chemical Mass
1	3257	RL	(138–139)	beta-lactokinin	Inhibitor of Angiotensin-Converting Enzyme (ACE) (EC 3.4.15.1) (MEROPS ID: XM02-001)	ACE inhibitor	287.1850	287.3480
2	7591	GF	(130–131)	ACE inhibitor	Inhibitor of Angiotensin-Converting Enzyme (ACE) (EC 3.4.15.1) (MEROPS ID: XM02-001)	ACE inhibitor	222.0890	222.2290
3	7599	GL	(183–184)	ACE inhibitor	Inhibitor of Angiotensin-converting enzyme (EC 3.4.15.1) (MEROPS ID: XM02-001)	ACE inhibitor	188.1050	188.2120
4	8561	GL	(183–184)	dipeptidyl peptidase IV inhibitor (DPP IV inhibitor)	Inhibitor of Dipeptidyl Peptidase IV (EC 3.4.14.5) (MEROPS ID: S09.003)	dipeptidyl peptidase IV inhibitor	188.1050	188.2120
5	8782	GF	(130–131)	dipeptidyl peptidase IV inhibitor (DPP IV inhibitor)	Inhibitor of Dipeptidyl Peptidase IV (EC 3.4.14.5) (MEROPS ID: S09.003)	dipeptidyl peptidase IV inhibitor	222.0890	222.2290
6	8886	RL	(138–139)	dipeptidyl peptidase IV inhibitor (DPP IV inhibitor)	Inhibitor of Dipeptidyl Peptidase IV (EC 3.4.14.5) (MEROPS ID: S09.003)	dipeptidyl peptidase IV inhibitor	287.1850	287.3480
7	9074	DF	(204–205)	ACE inhibitor	Inhibitor of Angiotensin-Converting Enzyme (ACE) (EC 3.4.15.1) (MEROPS ID: XM02-001)	ACE inhibitor	280.0949	280.2660

**Table A46:** *A_E_*, *DH_t_*, *W*, *B_E_* and *V* values from RubisCO of *Halophila stipulacea*. In silico enzymatic cleavage was carried out by using oligopeptidase F.

*DH_t_* (%)					
12.1951					
No.	Activity	*A_E_*	*W*	*B_E_*	*V*
1	ACE inhibitor	0.0194	0.0333	2.5006640432763E-5	0.00093451973448837
2	dipeptidyl peptidase IV inhibitor	0.0146	0.0224	1.8563339357632E-6	0.0086054864513139

**Table A47:** Bioactive peptides from RubisCO of *Halophila stipulacea*. In silico enzymatic cleavage was carried out by using proteinase P1 (lactocepin).

No.	Peptide ID	Sequence	Location	Name	Function	Activity	Monoisotopic Mass	Chemical Mass
1	3563	AY	(104–105)	ACE inhibitor	Inhibitor of Angiotensin-Converting Enzyme (ACE) (EC 3.4.15.1) (MEROPS ID: XM02-001)	ACE inhibitor	252.1000	252.2490
2	3563	AY	(147–148)	ACE inhibitor	Inhibitor of Angiotensin-Converting Enzyme (ACE) (EC 3.4.15.1) (MEROPS ID: XM02-001)	ACE inhibitor	252.1000	252.2490
3	4005	RA	(135–136)		Activation of ubiquitin-dependent proteolysis	activating ubiquitin-mediated proteolysis	245.1380	245.2670
4	7588	RA	(135–136)	ACE inhibitor	Inhibitor of Angiotensin-Converting Enzyme (ACE) (EC 3.4.15.1) (MEROPS ID: XM02-001)	ACE inhibitor	245.1380	245.2670
5	7591	GF	(12–13)	ACE inhibitor	Inhibitor of Angiotensin-Converting Enzyme (ACE) (EC 3.4.15.1) (MEROPS ID: XM02-001)	ACE inhibitor	222.0890	222.2290
6	7608	GV	(47–48)	ACE inhibitor	Inhibitor of Angiotensin-Converting Enzyme (ACE) (EC 3.4.15.1) (MEROPS ID: XM02-001)	ACE inhibitor	174.0890	174.1850
7	7866	AY	(104–105)	peptide from Okara protein	Peptide obtained by hydrolysis of Okara protein by use of enzymatic preparation Protease N.	antioxidative	252.1000	252.2490
8	7866	AY	(147–148)	peptide from Okara protein	Peptide obtained by hydrolysis of Okara protein by use of enzymatic preparation Protease N.	antioxidative	252.1000	252.2490
9	8526	RA	(135–136)	dipeptidyl peptidase IV inhibitor (DPP IV inhibitor)	Inhibitor of Dipeptidyl Peptidase IV (EC 3.4.14.5) (MEROPS ID: S09.003)	dipeptidyl peptidase IV inhibitor	245.1380	245.2670
10	8765	AY	(104–105)	dipeptidyl peptidase IV inhibitor (DPP IV inhibitor)	Inhibitor of Dipeptidyl Peptidase IV (EC 3.4.14.5) (MEROPS ID: S09.003)	dipeptidyl peptidase IV inhibitor	252.1000	252.2490
11	8765	AY	(147–148)	dipeptidyl peptidase IV inhibitor (DPP IV inhibitor)	Inhibitor of Dipeptidyl Peptidase IV (EC 3.4.14.5) (MEROPS ID: S09.003)	dipeptidyl peptidase IV inhibitor	252.1000	252.2490
12	8782	GF	(12–13)	dipeptidyl peptidase IV inhibitor (DPP IV inhibitor)	Inhibitor of Dipeptidyl Peptidase IV (EC 3.4.14.5) (MEROPS ID: S09.003)	dipeptidyl peptidase IV inhibitor	222.0890	222.2290
13	8786	GV	(47–48)	dipeptidyl peptidase IV inhibitor (DPP IV inhibitor)	Inhibitor of Dipeptidyl Peptidase IV (EC 3.4.14.5) (MEROPS ID: S09.003)	dipeptidyl peptidase IV inhibitor	174.0890	174.1850
14	8884	RI	(143–144)	dipeptidyl peptidase IV inhibitor (DPP IV inhibitor)	Inhibitor of Dipeptidyl Peptidase IV (EC 3.4.14.5) (MEROPS ID: S09.003)	dipeptidyl peptidase IV inhibitor	287.1850	287.3480
15	8895	SV	(114–115)	dipeptidyl peptidase IV inhibitor (DPP IV inhibitor)	Inhibitor of Dipeptidyl Peptidase IV (EC 3.4.14.5) (MEROPS ID: S09.003)	dipeptidyl peptidase IV inhibitor	204.1000	204.2130

**Table A48:** *A_E_*, *DH_t_*, *W*, *B_E_* and *V* values from RubisCO of *Halophila stipulacea*. In silico enzymatic cleavage carried out by using proteinase P1 (lactocepin).

*DH_t_* (%)					
41.4634					
No.	Activity	*A_E_*	*W*	*B_E_*	*V*
1	ACE inhibitor	0.0243	0.0417	0.00053029982441682	0.019817762103858
2	activating ubiquitin-mediated proteolysis	0.0049	0.3356	0	
3	antioxidative	0.0097	0.1332	0	0
4	dipeptidyl peptidase IV inhibitor	0.0340	0.0523	0	0

**Table A49:** Bioactive peptides from RubisCO of *Halophila stipulacea*. In silico enzymatic cleavage was carried out by using pepsin (pH >2).

No.	Peptide ID	Sequence	Location	Name	Function	Activity	Monoisotopic Mass	Chemical Mass
1	2754	PG	(46–47)	peptide regulating the stomach mucosal membrane activity	regulating the stomach mucosal membrane activity	regulating	172.0730	172.1690
2	3172	VA	(57–58)	dipeptidyl peptidase IV inhibitor (DPP IV inhibitor)	Inhibitor of Dipeptidyl Peptidase IV (EC 3.4.14.5) (MEROPS ID: S09.003)	dipeptidyl peptidase IV inhibitor	188.1050	188.2120
3	3172	VA	(92–93)	dipeptidyl peptidase IV inhibitor (DPP IV inhibitor)	Inhibitor of Dipeptidyl Peptidase IV (EC 3.4.14.5) (MEROPS ID: S09.003)	dipeptidyl peptidase IV inhibitor	188.1050	188.2120
4	3172	VA	(103–104)	dipeptidyl peptidase IV inhibitor (DPP IV inhibitor)	Inhibitor of Dipeptidyl Peptidase IV (EC 3.4.14.5) (MEROPS ID: S09.003)	dipeptidyl peptidase IV inhibitor	188.1050	188.2120
5	3257	RL	(138–139)	beta-lactokinin	Inhibitor of Angiotensin-Converting Enzyme (ACE) (EC 3.4.15.1) (MEROPS ID: XM02-001)	ACE inhibitor	287.1850	287.3480
6	3257	RL	(165–166)	beta-lactokinin	Inhibitor of Angiotensin-Converting Enzyme (ACE) (EC 3.4.15.1) (MEROPS ID: XM02-001)	ACE inhibitor	287.1850	287.3480
7	3285	PG	(46–47)	Antithrombotic peptide	Antithrombotic	antithrombotic	172.0730	172.1690
8	3380	RY	(81–82)	ACE inhibitor	Inhibitor of Angiotensin-Converting Enzyme (ACE) (EC 3.4.15.1) (MEROPS ID: XM02-001)	ACE inhibitor	337.1640	337.3580
9	3384	VF	(128–129)	ACE inhibitor	Inhibitor of Angiotensin-Converting Enzyme (ACE) (EC 3.4.15.1) (MEROPS ID: XM02-001)	ACE inhibitor	264.1360	264.3100
10	3460	PG	(46–47)	Prolyl endopeptidase inhibitor	Inhibitor of Prolyl Endopeptidase (PEP) (EC 3.4.21.26) (MEROPS ID: S09.001)	antiamnestic	172.0730	172.1690
11	3492	VY	(195–196)	ACE inhibitor from sake	Inhibitor of Angiotensin-Converting Enzyme (ACE) (EC 3.4.15.1) (MEROPS ID: XM02-001)	ACE inhibitor	280.1310	280.3030
12	4005	RA	(135–136)		Activation of ubiquitin-dependent proteolysis	activating ubiquitin-mediated proteolysis	245.1380	245.2670
13	4005	RA	(193–194)		Activation of ubiquitin-dependent proteolysis	activating ubiquitin-mediated proteolysis	245.1380	245.2670
14	7513	PL	(106–107)	ACE inhibitor from Alaskan pollack skin	Inhibitor of Angiotensin-converting enzyme (EC 3.4.15.1) (MEROPS ID: XM02-001)	ACE inhibitor	228.1360	228.2770
15	7558	VK	(17–18)	ACE inhibitor from buckwheat		ACE inhibitor	245.1620	245.3070
16	7562	IA	(100–101)	ACE inhibitor from soy hydrolysate		ACE inhibitor	202.1210	202.2390
17	7588	RA	(135–136)	ACE inhibitor	Inhibitor of Angiotensin-Converting Enzyme (ACE) (EC 3.4.15.1) (MEROPS ID: XM02-001)	ACE inhibitor	245.1380	245.2670
18	7588	RA	(193–194)	ACE inhibitor	Inhibitor of Angiotensin-Converting Enzyme (ACE) (EC 3.4.15.1) (MEROPS ID: XM02-001)	ACE inhibitor	245.1380	245.2670
19	7594	VG	(11–12)	ACE inhibitor	Inhibitor of Angiotensin-Converting Enzyme (ACE) (EC 3.4.15.1) (MEROPS ID: XM02-001)	ACE inhibitor	174.0890	174.1850
20	7594	VG	(125–126)	ACE inhibitor	Inhibitor of Angiotensin-Converting Enzyme (ACE) (EC 3.4.15.1) (MEROPS ID: XM02-001)	ACE inhibitor	174.0890	174.1850
21	7625	PG	(46–47)	ACE inhibitor	Inhibitor of Angiotensin-Converting Enzyme (ACE) (EC 3.4.15.1) (MEROPS ID: M02-001)	ACE inhibitor	172.0730	172.1690
22	7827	IE	(89–90)	ACE inhibitor		ACE inhibitor	260.1260	260.2760
23	7829	VE	(161–162)	ACE inhibitor	Inhibitor of Angiotensin-Converting Enzyme (ACE) (EC 3.4.15.1) (MEROPS ID: XM02-001)	ACE inhibitor	246.1100	246.2490
24	8224	VY	(195–196)	antioxidative peptide	free radical scavenging	antioxidative	280.1310	280.3030
25	8323	IL	(36–37)	Glucose uptake stimulating peptide	Glucose uptake stimulating	stimulating	244.1680	244.3200
26	8525	IA	(100–101)	dipeptidyl peptidase IV inhibitor (DPP IV inhibitor)	Inhibitor of Dipeptidyl Peptidase IV (EC 3.4.14.5) (MEROPS ID: S09.003)	dipeptidyl peptidase IV inhibitor	202.1210	202.2390
27	8526	RA	(135–136)	dipeptidyl peptidase IV inhibitor (DPP IV inhibitor)	Inhibitor of Dipeptidyl Peptidase IV (EC 3.4.14.5) (MEROPS ID: S09.003)	dipeptidyl peptidase IV inhibitor	245.1380	245.2670
28	8526	RA	(193–194)	dipeptidyl peptidase IV inhibitor (DPP IV inhibitor)	Inhibitor of Dipeptidyl Peptidase IV (EC 3.4.14.5) (MEROPS ID: S09.003)	dipeptidyl peptidase IV inhibitor	245.1380	245.2670
29	8560	SL	(78–79)	dipeptidyl peptidase IV inhibitor (DPP IV inhibitor)	Inhibitor of Dipeptidyl Peptidase IV (EC 3.4.14.5) (MEROPS ID: S09.003)	dipeptidyl peptidase IV inhibitor	218.1160	218.2400
30	8638	PL	(106–107)	dipeptidyl peptidase IV inhibitor (DPP IV inhibitor)	Inhibitor of Dipeptidyl Peptidase IV (EC 3.4.14.5) (MEROPS ID: S09.003)	dipeptidyl peptidase IV inhibitor	228.1360	228.2770
31	8685	WT	(68–69)	dipeptidyl peptidase IV inhibitor (DPP IV inhibitor)	Inhibitor of Dipeptidyl Peptidase IV (EC 3.4.14.5) (MEROPS ID: S09.003)	dipeptidyl peptidase IV inhibitor	305.1260	305.3180
32	8802	IL	(36–37)	dipeptidyl peptidase IV inhibitor (DPP IV inhibitor)	Inhibitor of Dipeptidyl Peptidase IV (EC 3.4.14.5) (MEROPS ID: S09.003)	dipeptidyl peptidase IV inhibitor	244.1680	244.3200
33	8805	IQ	(159–160)	dipeptidyl peptidase IV inhibitor (DPP IV inhibitor)	Inhibitor of Dipeptidyl Peptidase IV (EC 3.4.14.5) (MEROPS ID: S09.003)	dipeptidyl peptidase IV inhibitor	259.1420	259.2910
34	8855	PG	(46–47)	dipeptidyl peptidase IV inhibitor (DPP IV inhibitor)	Inhibitor of Dipeptidyl Peptidase IV (EC 3.4.14.5) (MEROPS ID: S09.003)	dipeptidyl peptidase IV inhibitor	172.0730	172.1690
35	8858	PK	(180–181)	dipeptidyl peptidase IV inhibitor (DPP IV inhibitor)	Inhibitor of Dipeptidyl Peptidase IV (EC 3.4.14.5) (MEROPS ID: S09.003)	dipeptidyl peptidase IV inhibitor	243.1460	243.2910
36	8882	RG	(200–201)	dipeptidyl peptidase IV inhibitor (DPP IV inhibitor)	Inhibitor of Dipeptidyl Peptidase IV (EC 3.4.14.5) (MEROPS ID: S09.003)	dipeptidyl peptidase IV inhibitor	231.1220	231.2400
37	8886	RL	(138–139)	dipeptidyl peptidase IV inhibitor (DPP IV inhibitor)	Inhibitor of Dipeptidyl Peptidase IV (EC 3.4.14.5) (MEROPS ID: S09.003)	dipeptidyl peptidase IV inhibitor	287.1850	287.3480
38	8886	RL	(165–166)	dipeptidyl peptidase IV inhibitor (DPP IV inhibitor)	Inhibitor of Dipeptidyl Peptidase IV (EC 3.4.14.5) (MEROPS ID: S09.003)	dipeptidyl peptidase IV inhibitor	287.1850	287.3480
39	8894	SK	(149–150)	dipeptidyl peptidase IV inhibitor (DPP IV inhibitor)	Inhibitor of Dipeptidyl Peptidase IV (EC 3.4.14.5) (MEROPS ID: S09.003)	dipeptidyl peptidase IV inhibitor	233.1260	233.2540
40	8916	VE	(161–162)	dipeptidyl peptidase IV inhibitor (DPP IV inhibitor)	Inhibitor of Dipeptidyl Peptidase IV (EC 3.4.14.5) (MEROPS ID: S09.003)	dipeptidyl peptidase IV inhibitor	246.1100	246.2490
41	8917	VF	(128–129)	dipeptidyl peptidase IV inhibitor (DPP IV inhibitor)	Inhibitor of Dipeptidyl Peptidase IV (EC 3.4.14.5) (MEROPS ID: S09.003)	dipeptidyl peptidase IV inhibitor	264.1360	264.3100
42	8918	VG	(11–12)	dipeptidyl peptidase IV inhibitor (DPP IV inhibitor)	Inhibitor of Dipeptidyl Peptidase IV (EC 3.4.14.5) (MEROPS ID: S09.003)	dipeptidyl peptidase IV inhibitor	174.0890	174.1850
43	8918	VG	(125–126)	dipeptidyl peptidase IV inhibitor (DPP IV inhibitor)	Inhibitor of Dipeptidyl Peptidase IV (EC 3.4.14.5) (MEROPS ID: S09.003)	dipeptidyl peptidase IV inhibitor	174.0890	174.1850
44	8921	VK	(17–18)	dipeptidyl peptidase IV inhibitor (DPP IV inhibitor)	Inhibitor of Dipeptidyl Peptidase IV (EC 3.4.14.5) (MEROPS ID: S09.003)	dipeptidyl peptidase IV inhibitor	245.1620	245.3070
45	8927	VT	(115–116)	dipeptidyl peptidase IV inhibitor (DPP IV inhibitor)	Inhibitor of Dipeptidyl Peptidase IV (EC 3.4.14.5) (MEROPS ID: S09.003)	dipeptidyl peptidase IV inhibitor	218.1150	218.2380
46	8929	VY	(195–196)	dipeptidyl peptidase IV inhibitor (DPP IV inhibitor)	Inhibitor of Dipeptidyl Peptidase IV (EC 3.4.14.5) (MEROPS ID: S09.003)	dipeptidyl peptidase IV inhibitor	280.1310	280.3030
47	9079	IL	(36–37)	ACE inhibitor	Inhibitor of Angiotensin-Converting Enzyme (ACE) (EC 3.4.15.1) (MEROPS ID: XM02-001)	ACE inhibitor	244.1680	244.3200

**Table A50:** *A_E_*, *DH_t_*, *W*, *B_E_* and *V* values from RubisCO of *Halophila stipulacea*. In silico enzymatic cleavage carried out by using pepsin (pH >2).

*DH_t_* (%)					
70.7317					
No.	Activity	*A_E_*	*W*	*B_E_*	*V*
1	regulating	0.0049	0.3356	0	
2	dipeptidyl peptidase IV inhibitor	0.1165	0.1791	9.8559287499226E-5	0.45689549540936
3	ACE inhibitor	0.0777	0.1334	0.002215755283035	0.082804687193409
4	antithrombotic	0.0049	0.5052	0	
5	antiamnestic	0.0049	0.5052	0	
6	activating ubiquitin-mediated proteolysis	0.0097	0.6644	0	
7	antioxidative	0.0049	0.0673	0	0
8	stimulating	0.0049	0.1441	0	

**Table A51:** Bioactive peptides from RubisCO of *Halophila stipulacea*. In silico enzymatic cleavage was carried out by using cocolysin.

No.	Peptide ID	Sequence	Location	Name	Function	Activity	Monoisotopic Mass	Chemical Mass
1	3522	IPP	(144–146)	ACE inhibitor (from bovine b-CN)		ACE inhibitor	325.1880	325.3940
2	3666	YP	(105–106)	ACE inhibitor	Inhibitor of Angiotensin-Converting Enzyme (ACE) (EC 3.4.15.1) (MEROPS ID: XM02-001)	ACE inhibitor	278.1150	278.2870
3	7600	AG	(53–54)	ACE inhibitor	Inhibitor of Angiotensin-Converting Enzyme (ACE) (EC 3.4.15.1) (MEROPS ID: XM02-001)	ACE inhibitor	146.0580	146.1310
4	7605	FG	(129–130)	ACE inhibitor	Inhibitor of Angiotensin-Converting Enzyme (ACE) (EC 3.4.15.1) (MEROPS ID: XM02-001)	ACE inhibitor	222.0890	222.2290
5	7619	LG	(182–183)	ACE inhibitor	Inhibitor of Angiotensin-converting enzyme (EC 3.4.15.1) (MEROPS ID: XM02-001)	ACE inhibitor	188.1050	188.2120
6	7697	YK	(20–21)	ACE inhibitor from wakame		ACE inhibitor	309.1570	309.3440
7	8521	YP	(105–106)	dipeptidyl peptidase IV inhibitor (DPP IV inhibitor)	Inhibitor of Dipeptidyl Peptidase IV (EC 3.4.14.5) (MEROPS ID: S09.003)	dipeptidyl peptidase IV inhibitor	278.1150	278.2870
8	8760	AG	(53–54)	dipeptidyl peptidase IV inhibitor (DPP IV inhibitor)	Inhibitor of Dipeptidyl Peptidase IV (EC 3.4.14.5) (MEROPS ID: S09.003)	dipeptidyl peptidase IV inhibitor	146.0580	146.1310
9	8764	AV	(56–57)	dipeptidyl peptidase IV inhibitor (DPP IV inhibitor)	Inhibitor of Dipeptidyl Peptidase IV (EC 3.4.14.5) (MEROPS ID: S09.003)	dipeptidyl peptidase IV inhibitor	188.1050	188.2120
10	8764	AV	(194–195)	dipeptidyl peptidase IV inhibitor (DPP IV inhibitor)	Inhibitor of Dipeptidyl Peptidase IV (EC 3.4.14.5) (MEROPS ID: S09.003)	dipeptidyl peptidase IV inhibitor	188.1050	188.2120
11	8779	FQ	(13–14)	dipeptidyl peptidase IV inhibitor (DPP IV inhibitor)	Inhibitor of Dipeptidyl Peptidase IV (EC 3.4.14.5) (MEROPS ID: S09.003)	dipeptidyl peptidase IV inhibitor	293.1260	293.3080
12	8824	LT	(22–23)	dipeptidyl peptidase IV inhibitor (DPP IV inhibitor)	Inhibitor of Dipeptidyl Peptidase IV (EC 3.4.14.5) (MEROPS ID: S09.003)	dipeptidyl peptidase IV inhibitor	232.1310	232.2650
13	8937	YH	(87–88)	dipeptidyl peptidase IV inhibitor (DPP IV inhibitor)	Inhibitor of Dipeptidyl Peptidase IV (EC 3.4.14.5) (MEROPS ID: S09.003)	dipeptidyl peptidase IV inhibitor	318.1210	318.3120
14	8939	YK	(20–21)	dipeptidyl peptidase IV inhibitor (DPP IV inhibitor)	Inhibitor of Dipeptidyl Peptidase IV (EC 3.4.14.5) (MEROPS ID: S09.003)	dipeptidyl peptidase IV inhibitor	309.1570	309.3440
15	8946	YV	(102–103)	dipeptidyl peptidase IV inhibitor (DPP IV inhibitor)	Inhibitor of Dipeptidyl Peptidase IV (EC 3.4.14.5) (MEROPS ID: S09.003)	dipeptidyl peptidase IV inhibitor	280.1310	280.3030
16	8951	AV	(56–57)	ACE inhibitor	Inhibitor of Angiotensin-Converting Enzyme (ACE) (EC 3.4.15.1) (MEROPS ID: XM02-001)	ACE inhibitor	188.1050	188.2120
17	8951	AV	(194–195)	ACE inhibitor	Inhibitor of Angiotensin-Converting Enzyme (ACE) (EC 3.4.15.1) (MEROPS ID: XM02-001)	ACE inhibitor	188.1050	188.2120
18	9076	FQ	(13–14)	ACE inhibitor	Inhibitor of Angiotensin-Converting Enzyme (ACE) (EC 3.4.15.1) (MEROPS ID: XM02-001)	ACE inhibitor	293.1260	293.3080
19	9077	YV	(102–103)	ACE inhibitor	Inhibitor of Angiotensin-Converting Enzyme (ACE) (EC 3.4.15.1) (MEROPS ID: XM02-001)	ACE inhibitor	280.1310	280.3030
20	9087	YH	(87–88)	ACE inhibitor	Inhibitor of Angiotensin-Converting Enzyme (ACE) (EC 3.4.15.1) (MEROPS ID: XM02-001)	ACE inhibitor	318.1210	318.3120
21	9213	LR	(134–135)	ACE inhibitor	Inhibitor of Angiotensin-Converting Enzyme (ACE) (EC 3.4.15.1) (MEROPS ID: M02-001)	ACE inhibitor	287.1850	287.3480
22	9213	LR	(137–138)	ACE inhibitor	Inhibitor of Angiotensin-Converting Enzyme (ACE) (EC 3.4.15.1) (MEROPS ID: M02-001)	ACE inhibitor	287.1850	287.3480
23	9213	LR	(142–143)	ACE inhibitor	Inhibitor of Angiotensin-Converting Enzyme (ACE) (EC 3.4.15.1) (MEROPS ID: M02-001)	ACE inhibitor	287.1850	287.3480

**Table A52:** *A_E_*, *DH_t_*, *W*, *B_E_* and *V* values from RubisCO of *Halophila stipulacea*. In silico enzymatic cleavage was carried out by using cocolysin.

*DH_t_* (%)					
30.2439					
No.	Activity	*A_E_*	*W*	*B_E_*	*V*
1	ACE inhibitor	0.0680	0.1167	0.0021410562742961	0.080013120769247
2	dipeptidyl peptidase IV inhibitor	0.0437	0.0672	1.5313466662583E-6	0.0070989290961449

**Table A53:** Bioactive peptides from RubisCO of *Halophila stipulacea*. In silico enzymatic cleavage was carried out by using subtilisin.

No.	Peptide ID	Sequence	Location	Name	Function	Activity	Monoisotopic Mass	Chemical Mass
1	3257	RL	(138–139)	beta-lactokinin	Inhibitor of Angiotensin-Converting Enzyme (ACE) (EC 3.4.15.1) (MEROPS ID: XM02-001)	ACE inhibitor	287.1850	287.3480
2	3384	VF	(128–129)	ACE inhibitor	Inhibitor of Angiotensin-Converting Enzyme (ACE) (EC 3.4.15.1) (MEROPS ID: XM02-001)	ACE inhibitor	264.1360	264.3100
3	3486	VW	(71–72)	ACE inhibitor from sake lees	Inhibitor of Angiotensin-Converting Enzyme (ACE) (EC 3.4.15.1) (MEROPS ID: XM02-001)	ACE inhibitor	303.1470	303.3460
4	3492	VY	(195–196)	ACE inhibitor from sake	Inhibitor of Angiotensin-Converting Enzyme (ACE) (EC 3.4.15.1) (MEROPS ID: XM02-001)	ACE inhibitor	280.1310	280.3030
5	3546	VAY	(103–105)	ACE inhibitor	Inhibitor of Angiotensin-Converting Enzyme (ACE) (EC 3.4.15.1) (MEROPS ID: XM02-001)	ACE inhibitor	351.1680	351.3820
6	7513	PL	(106–107)	ACE inhibitor from Alaskan pollack skin	Inhibitor of Angiotensin-converting enzyme (EC 3.4.15.1) (MEROPS ID: XM02-001)	ACE inhibitor	228.1360	228.2770
7	7591	GF	(130–131)	ACE inhibitor	Inhibitor of Angiotensin-Converting Enzyme (ACE) (EC 3.4.15.1) (MEROPS ID: XM02-001)	ACE inhibitor	222.0890	222.2290
8	7599	GL	(183–184)	ACE inhibitor	Inhibitor of Angiotensin-converting enzyme (EC 3.4.15.1) (MEROPS ID: XM02-001)	ACE inhibitor	188.1050	188.2120
9	7693	KL	(21–22)	ACE inhibitor from wakame		ACE inhibitor	259.1780	259.3340
10	8219	TY	(23–24)	antioxidative peptide		antioxidative	282.1100	282.2750
11	8224	VY	(195–196)	antioxidative peptide	free radical scavenging	antioxidative	280.1310	280.3030
12	8461	VW	(71–72)	Antioxidant peptide from marine bivalve (Mactra veneriformis)	Antioxidant	antioxidative	303.1470	303.3460
13	8561	GL	(183–184)	dipeptidyl peptidase IV inhibitor (DPP IV inhibitor)	Inhibitor of Dipeptidyl Peptidase IV (EC 3.4.14.5) (MEROPS ID: S09.003)	dipeptidyl peptidase IV inhibitor	188.1050	188.2120
14	8638	PL	(106–107)	dipeptidyl peptidase IV inhibitor (DPP IV inhibitor)	Inhibitor of Dipeptidyl Peptidase IV (EC 3.4.14.5) (MEROPS ID: S09.003)	dipeptidyl peptidase IV inhibitor	228.1360	228.2770
15	8782	GF	(130–131)	dipeptidyl peptidase IV inhibitor (DPP IV inhibitor)	Inhibitor of Dipeptidyl Peptidase IV (EC 3.4.14.5) (MEROPS ID: S09.003)	dipeptidyl peptidase IV inhibitor	222.0890	222.2290
16	8886	RL	(138–139)	dipeptidyl peptidase IV inhibitor (DPP IV inhibitor)	Inhibitor of Dipeptidyl Peptidase IV (EC 3.4.14.5) (MEROPS ID: S09.003)	dipeptidyl peptidase IV inhibitor	287.1850	287.3480
17	8910	TS	(77–78)	dipeptidyl peptidase IV inhibitor (DPP IV inhibitor)	Inhibitor of Dipeptidyl Peptidase IV (EC 3.4.14.5) (MEROPS ID: S09.003)	dipeptidyl peptidase IV inhibitor	206.0790	206.1850
18	8910	TS	(120–121)	dipeptidyl peptidase IV inhibitor (DPP IV inhibitor)	Inhibitor of Dipeptidyl Peptidase IV (EC 3.4.14.5) (MEROPS ID: S09.003)	dipeptidyl peptidase IV inhibitor	206.0790	206.1850
19	8911	TT	(69–70)	dipeptidyl peptidase IV inhibitor (DPP IV inhibitor)	Inhibitor of Dipeptidyl Peptidase IV (EC 3.4.14.5) (MEROPS ID: S09.003)	dipeptidyl peptidase IV inhibitor	220.0940	220.2100
20	8914	TY	(23–24)	dipeptidyl peptidase IV inhibitor (DPP IV inhibitor)	Inhibitor of Dipeptidyl Peptidase IV (EC 3.4.14.5) (MEROPS ID: S09.003)	dipeptidyl peptidase IV inhibitor	282.1100	282.2750
21	8917	VF	(128–129)	dipeptidyl peptidase IV inhibitor (DPP IV inhibitor)	Inhibitor of Dipeptidyl Peptidase IV (EC 3.4.14.5) (MEROPS ID: S09.003)	dipeptidyl peptidase IV inhibitor	264.1360	264.3100
22	8926	VS	(42–43)	dipeptidyl peptidase IV inhibitor (DPP IV inhibitor)	Inhibitor of Dipeptidyl Peptidase IV (EC 3.4.14.5) (MEROPS ID: S09.003)	dipeptidyl peptidase IV inhibitor	204.1000	204.2130
23	8928	VW	(71–72)	dipeptidyl peptidase IV inhibitor (DPP IV inhibitor)	Inhibitor of Dipeptidyl Peptidase IV (EC 3.4.14.5) (MEROPS ID: S09.003)	dipeptidyl peptidase IV inhibitor	303.1470	303.3460
24	8929	VY	(195–196)	dipeptidyl peptidase IV inhibitor (DPP IV inhibitor)	Inhibitor of Dipeptidyl Peptidase IV (EC 3.4.14.5) (MEROPS ID: S09.003)	dipeptidyl peptidase IV inhibitor	280.1310	280.3030
25	9071	IAY	(100–102)	ACE inhibitor	Inhibitor of Angiotensin-Converting Enzyme (ACE) (EC 3.4.15.1) (MEROPS ID: M02-001)	ACE inhibitor	365.1840	365.4090
26	9074	DF	(204–205)	ACE inhibitor	Inhibitor of Angiotensin-Converting Enzyme (ACE) (EC 3.4.15.1) (MEROPS ID: XM02-001)	ACE inhibitor	280.0949	280.2660

**Table A54:** *A_E_*, *DH_t_*, *W*, *B_E_* and *V* values from RubisCO of *Halophila stipulacea*. In silico enzymatic cleavage was carried out by using subtilisin.

*DH_t_* (%)					
29.2683					
No.	Activity	*A_E_*	*W*	*B_E_*	*V*
1	ACE inhibitor	0.0534	0.0917	0.0055038387628308	0.20568320455268
2	antioxidative	0.0146	0.2005	0	0
3	dipeptidyl peptidase IV inhibitor	0.0583	0.0896	1.8563339357632E-6	0.0086054864513139

**Table A55:** Bioactive peptides from RubisCO of *Halophila stipulacea*. In silico enzymatic cleavage was carried out by using V-8 protease (Glutamyl endopeptidase).

No.	Peptide ID	Sequence	Location	Name	Function	Activity	Monoisotopic Mass	Chemical Mass
1	8898	TD	(34–35)	dipeptidyl peptidase IV inhibitor (DPP IV inhibitor)	Inhibitor of Dipeptidyl Peptidase IV (EC 3.4.14.5) (MEROPS ID: S09.003)	dipeptidyl peptidase IV inhibitor	234.0739	234.1940
2	8934	YE	(29–30)	dipeptidyl peptidase IV inhibitor (DPP IV inhibitor)	Inhibitor of Dipeptidyl Peptidase IV (EC 3.4.14.5) (MEROPS ID: S09.003)	dipeptidyl peptidase IV inhibitor	310.1050	310.2860
3	9078	YE	(29–30)	ACE inhibitor	Inhibitor of Angiotensin-Converting Enzyme (ACE) (EC 3.4.15.1) (MEROPS ID: XM02-001)	ACE inhibitor	310.1050	310.2860

**Table A56:** *A_E_*, *DH_t_*, *W*, *B_E_* and *V* values from RubisCO of *Halophila stipulacea*. In silico enzymatic cleavage was carried out by using V-8 protease (Glutamyl endopeptidase).

*DH_t_* (%)					
11.7073					
No.	Activity	*A_E_*	*W*	*B_E_*	*V*
1	dipeptidyl peptidase IV inhibitor	0.0097	0.0149	0	0
2	ACE inhibitor	0.0049	0.0084	7.6943555746376E-6	0.0002875447082947
